# Bridging the gaps in statistical models of protein alignment

**DOI:** 10.1093/bioinformatics/btac246

**Published:** 2022-06-27

**Authors:** Dinithi Sumanaweera, Lloyd Allison, Arun S Konagurthu

**Affiliations:** Department of Data Science and Artificial Intelligence, Faculty of Information Technology, Monash University, Clayton, VIC 3800, Australia; Department of Data Science and Artificial Intelligence, Faculty of Information Technology, Monash University, Clayton, VIC 3800, Australia; Department of Data Science and Artificial Intelligence, Faculty of Information Technology, Monash University, Clayton, VIC 3800, Australia

## Abstract

**Summary:**

Sequences of proteins evolve by accumulating substitutions together with insertions and deletions (indels) of amino acids. However, it remains a common practice to disconnect substitutions and indels, and infer approximate models for each of them separately, to quantify sequence relationships. Although this approach brings with it computational convenience (which remains its primary motivation), there is a dearth of attempts to unify and model them systematically and together. To overcome this gap, this article demonstrates how a complete statistical model quantifying the evolution of pairs of aligned proteins can be constructed using a time-parameterized substitution matrix and a time-parameterized alignment state machine. Methods to derive all parameters of such a model from any benchmark collection of aligned protein sequences are described here. This has not only allowed us to generate a unified statistical model for each of the nine widely used substitution matrices (PAM, JTT, BLOSUM, JO, WAG, VTML, LG, MIQS and PFASUM), but also resulted in a new unified model, MMLSUM. Our underlying methodology measures the Shannon information content using each model to explain losslessly any given collection of alignments, which has allowed us to quantify the performance of all the above models on six comprehensive alignment benchmarks. Our results show that MMLSUM results in a new and clear overall best performance, followed by PFASUM, VTML, BLOSUM and MIQS, respectively, amongst the top five. We further analyze the statistical properties of MMLSUM model and contrast it with others.

**Supplementary information:**

Supplementary data are available at *Bioinformatics* online.

## 1 Introduction

Comparing and inferring relationships between protein sequences is a challenging task and, when done properly, provides a powerful way to reason about the macromolecular consequences of evolution (Lesk, 2016). Many biological studies rely on identifying homologous relationships between proteins. The details of those relationships are represented as correspondences (alignment) between subsets of their amino acids. Each such correspondence suggests the divergence of the observed amino acids arising from a common locus within the ancestral genome.

Although sequence comparison is a mature field, the fidelity of the relationships evinced by modern homology detection and alignment programs remains a function of the underlying models they employ to evaluate hypothesized relationships. Most programs utilize a substitution matrix to quantify amino acid interchanges. These matrices are often parameterized on a numeric value that accounts for the extent of divergence/similarity between protein sequences [e.g. PAM-250 (Dayhoff *et al.*, 1978) and BLOSUM-62 (Henikoff and Henikoff, 1992)]. Separate gap-open and gap-extension penalties are widely felt to give plausible and sufficiently flexible approximations to quantify indels. However, mathematically reconciling the quantification of substitutions with that of indels remains contentious. Often the issue is simply avoided: previous studies have shown that the choices of which substitution matrix to use, at what threshold of divergence/similarity, and with what gap penalty values, remain anecdotal, sometimes empirical, if not fully arbitrary (Do *et al.*, 2005; Sumanaweera *et al.*, 2019; Vingron and Waterman, 1994).

Several notable works in the literature (Benner *et al.*, 1993; Blake and Cohen, 2001; Chang and Benner, 2004; Gonnet *et al.*, 1992; Holmes, 1998; 2017; Pang *et al.*, 2005; Pascarella and Argos, 1992; Rivas, 2005; Rivas and Eddy, 2015; Vogt *et al.*, 1995) highlight the importance of handling substitutions and indels jointly, via experiments that explore the relationship between amino acid correspondences and gap lengths with respect to evolutionary divergence. For instance, Holmes (1998) notably discusses the evolutionary time-dependent substitution models and gap models in hidden Markov model-based protein alignment, whereas Rivas (2005); Rivas and Eddy (2015) introduced explicit probabilistic evolutionary models to obtain time-dependent affine gap scores. Despite these noteworthy efforts, the field continues to lack a single formal *unified* framework to derive time-dependent substitutions and gap models. Further, an evaluation of the performance of existing substitution matrices is hampered by the fact that different sequence comparison programs yield conflicting results (Barton and Sternberg, 1987; Blake and Cohen, 2001; Do *et al.*, 2005; Löytynoja and Goldman, 2008; Vingron and Waterman, 1994). Thus, as the field stands, it lacks an objective framework to assess how well the commonly used substitution matrices perform for the task of comparison, without being impeded by *ad hoc* parameter choices.

In this article, we describe an unsupervised probabilistic and information-theoretic framework that uniquely allows us to: (i) compare the performance of existing substitution matrices, in terms of the Shannon information content (Shannon, 1948), without the need for any parameter-fiddling, (ii) infer improved stochastic Markov models of substitution that demonstrably outperform existing substitution matrices and (iii) infer time-parameterized three-state alignment models accompanying the above Markov substitution models, which together provide a unified way to address both amino acid indels and substitutions. Specifically, for any given collection of benchmark alignments, our framework uses the Bayesian Minimum Message Length (MML) criterion (Wallace and Boulton, 1968; Wallace and Freeman, 1987) to estimate the Shannon information content (Shannon, 1948) of that collection, measured in bits. The Shannon information provides a theoretical lower bound to the codeword length required to encode losslessly (i.e. compress) any given event within a message of transmission, computed using that event’s probability. Accordingly, the Shannon information content of an alignment collection is computed as the shortest encoding length required to compress losslessly all sequence pairs in that collection using their stated alignment relationships. This measure is based on rigorous probabilistic models that capture protein evolution (both in terms of substitutions *and* indels).

Parameters of the models are inferred unsupervised (i.e. automatically) by maximizing the lossless compression that can be gained from the collection. In general, Shannon information is a fundamental and measurable property of data, and has had effective use in studies involving biological macromolecules (Adami, 2004; Adjeroh and Nan, 2006; Allison and Yee, 1990; Cao *et al.*, 2007; Hategan and Tabus, 2004; Strait and Dewey, 1996). During model inference, MML provides an explicit representation of all parameters and their complexities, by taking into account the optimal precision and the complexity involved with every parameter statement, unlike other common methods such as maximum likelihood estimation (Allison, 2018; Wallace, 2005). MML has been applied to DNA/protein sequence alignment and phylogeny inference (Allison *et al.*, 1992; Allison and Wallace, 1994; Powell *et al.*, 2004; Yee and Allison, 1993), and protein sequence alignment through our recent work (Sumanaweera *et al.*, 2018, 2019).

Central to our information-theoretic framework is a time-parameterized stochastic matrix which gives the probabilities of interchanges between amino acids as a function of a Markov time parameter. This matrix works in concert with a set of time-parameterized Dirichlet probability distributions, learnt unsupervised from any stated alignment collection. Each (time-parameterized) Dirichlet distribution is used to model the corresponding alignment three-state machine over match, insert and delete states, and quantify the descriptive complexity of any alignment in information terms. Importantly, this formal handling of alignment complexity using these models rectifies the *ad hoc* considerations of gap penalties, commonly used by sequence alignment methods.

Crucially, all probabilistic parameters including the stochastic matrix in this framework can be automatically inferred. As a result, we have been able to infer a new and demonstrably improved model of protein evolution. Our framework also facilitates a conversion of existing substitution matrices to their corresponding Markov matrices with high-fidelity, even those that do not explicitly model amino acid interchanges as a Markov process [We note that there have been only a few varying attempts at such a conversion (Rivas, 2005; Veerassamy *et al.*, 2003)]. This provides a direct way to objectively compare the performance of various substitution matrices, without any need for parameter-tuning. To the best of our knowledge, the above mentioned features are unique to the work presented here. Our method of MML-based model estimation is described in Section 2.

The inferred time-parameterized models along with all other models compared here are freely available for use with the latest version of seqMMLigner (Sumanaweera *et al.*, 2019) program for pairwise sequence comparison.

## 2 Materials and methods

### 2.1 Introduction to minimum message length inference

The Minimum Message Length (MML) principle (Allison, 2018; Wallace, 2005; Wallace and Boulton, 1968) is a powerful technique to infer reliable hypotheses (models, theories) from observed data. MML is an information-theoretic criterion that, in its mechanics, combines Bayesian inference (Bayes, 1763) with lossless data compression. Formally, the joint probability of any hypothesis *H* and data *D* is given by: Pr(H,D)=Pr(H)Pr(D|H)=Pr(D)Pr(H|D) as per the Bayes theorem. Commonly, model inference depends on identifying suitable hypotheses based on posterior probability (i.e. Pr(H|D)). Separately, Shannon’s Mathematical Theory of Communication (Shannon, 1948) quantifies the amount of information in any event *E* that occurs with a probability of Pr(E) as: $I(E)=-{\;\text{log}\;}_{2}\text{Pr}(E)\;\text{bits}$. *I*(*E*) can be understood as the minimum lossless encoding length required to communicate the event E. Accordingly, the joint probability Pr(H,D) can be expressed in terms of Shannon information content as:

(1) I(H,D)=I(H)+I(D|H)=I(D)+I(H|D) bits

This relationship can be rationalized as the length of a two-part message required to communicate the hypothesis *H* and the data *D* given *H*, as a notional communication between a transmitter and receiver. In this formulation, the transmitter losslessly encodes the hypothesis *H* which takes *I*(*H*) bits to state, followed by the data *D* given the stated hypothesis H, taking another I(D|H) bits to state. Note, for any *H* and *D*, *I*(*H*) and I(D|H) should be accurately estimated, and we carry out this estimation using the well-established technique of Wallace and Freeman (1987) from the statistical-learning literature. Note, one of the important aspects of MML is its consideration of all model complexities—the statement (i.e. lossless encoding) of every parameter estimate to its optimum precision, which in turn ensures an honest evaluation of the model at hand.

Many attractive properties emerge from the MML formulation, but most useful here is the observation that the difference in message lengths between any pair of competing hypotheses (say *H*_1_ and *H*_2_) gives the posterior log-odds ratio as: $I(H_{1},D)-I(H_{2},D)=-{\;\text{log}\;}_{2}(\text{Pr}(D)\text{Pr}(H_{1}|D))+{\;\text{log}\;}_{2}(\text{Pr}(D)\text{Pr}(H_{2}|D))={\;\text{log}\;}_{2}\left(\frac{\text{Pr}(H_{2}|D)}{\text{Pr}(H_{1}|D)}\right)$ bits. This allows competing hypotheses to be objectively compared and the best one to be reliably chosen. See Allison (2018) and Wallace (2005) for further mathematical details on model selection.

### 2.2 Formulating the description of a protein alignment dataset using the MML framework

In this work, the observed data **D** denotes any (benchmark) dataset of aligned protein sequences. Formally, it is composed of pairs of amino acid sequences and their *given* alignments $D=\{\langle A_{1},S_{1},T_{1}\rangle ,\langle A_{2},S_{2},T_{2}\rangle ,\ldots ,\langle A_{\left|D\right|},S_{\left|D\right|},T_{\left|D\right|}\rangle \}$, where each *S_i_* and each *T_i_* are sequences over the alphabet of 20 amino acids, and $A_{i}$ represents their given alignment relationship specified as a 3-state string over {match, insert, delete} states. Note that each alignment $A_{i}$ is a part of the observed data, coming from structural alignments or from some set of benchmark alignments.

A hypothesis *H* that losslessly explains the above data **D** is composed of the following statistical models (and we emphasize that all the models shown below are automatically inferred from any given **D**, as those that are optimal under the MML criterion):

1. A time-parameterized stochastic Markov matrix M(t) which is used to encode losslessly the corresponding pairs of amino acids in $\langle S_{i},T_{i}\rangle$ that are under ‘match’ states in $A_{i}$ at time *t*. Note, this matrix can either be optimally inferred under the MML criterion from the collection **D**, or for comparison purposes, be any existing substitution matrix.
2. A multinomial model **P**, of the 20 amino acids, used to encode losslessly the amino acids in the unaligned regions of $\langle S_{i},T_{i}\rangle$ [i.e. those under insert or delete (indel) states in $A_{i}$]. Estimates of **P** are optimally inferred using MML from the indel regions observed in the alignments of the collection **D** [Note, for a comparison, we also explore two other choices for **P**: one derived from the stationary distribution of **M**, and the other one derived from a data source independent of **D** (refer Supplementary Section S2.2)].
3. A set of automatically inferred Dirichlet parameters α, each one specifying a Dirichlet distribution for a specific value of divergence ‘time’ *t*, to be used in conjunction with M(t); the alignment 3-state machine’s transition probabilities $\Theta_{i}$ that are inferred optimally for any alignment $A_{i}$ are encoded using one of the time-dependent Dirichlet distributions. These transition probabilities in turn are used to encode losslessly a 3-state alignment string $A_{i}$.
4. Finally, the set of automatically inferred time parameters $\tau =\{t_{1},t_{2},\ldots ,t_{N}\}$, one for each sequence-pair in **D** using the above models. Each *t_i_* captures the divergence of corresponding sequence-pairs $\langle S_{i},T_{i}\rangle$, where *t_i_* can be interpreted as the length of the Markov chain by which their amino acids are related using the above models.

Using Equation (1), this framework allows the estimation of the total Shannon information content in the hypothesis *H* and data **D** as a summation of individual Shannon information terms:

(2) $$\begin{array}{c} I(H,D)=I(M)+I(P)+I(\alpha )+\sum_{i=1}^{\left|D\right|}I(t_{i})+I(\Theta_{i}|\alpha ,t_{i})\\ +\;I(A_{i}|\Theta_{i},t_{i})+I(\langle S_{i},T_{i}\rangle |A_{i},M,P,t_{i})\;\text{bits} \end{array}$$

where I(M) is the lossless encoding (i.e. statement) length of Matrix **M** that models matched amino acid regions of **D**; I(P) is the statement length of probability estimates **P** to model indel regions of **D**; I(α) is the statement length of inferred time-dependent Dirichlet parameters; $I(t_{i})$ is the statement length of inferred time *t_i_* of a sequence-pair $\langle S_{i},T_{i}\rangle$ given its alignment $A_{i}$; $I(\Theta_{i}|\alpha ,t_{i})$ is the statement length of alignment 3-state machine parameters inferred on each $A_{i}$; $I(A_{i}|\Theta_{i},t_{i})$ is the statement length of each $A_{i}$; and $I(\langle S_{i},T_{i}\rangle |A_{i},M,P,t_{i})$ is the statement length of explaining all amino acids in the sequence-pair. The above models and their MML estimation are described in the following sections (see Supplementary Section S1).

This work utilizes the six alignment benchmarks described in Supplementary Section S4 to validate the framework introduced above. Each benchmark individually provides a source collection **D** of pairs of sequences and their given alignment relationships. **D** is losslessly compressed under the minimum message length (MML) criterion defined by Equation (2). New stochastic models of amino acid exchanges are automatically inferred on the above benchmarks and performance compared (in Shannon information terms) to the popularly used substitution matrices (historical and recent matrices including five Markov models and four non-Markov models) described in Supplementary Section S4, without the necessity to hand-tune parameters (as demonstrated in Section 3).

### 2.3 Stochastic matrix M(t) to model amino acids in the matched regions

Amino acid interchanges are modeled by a Markov chain (Norris and Norris, 1998) defined over the state space of 20 amino acids. That means, the probabilities of transitions between any pair of amino acids is represented as a stochastic matrix M(t). For any time *t *>* *0, if an amino acid *a*_0_ (at time *t *=* *0) undergoes the following chain of interchanges ($a_{0}\to a_{1}\to \ldots \to a_{t-1}\to a_{t}$), the Markov process ensures that the state of the amino acid *a_t_* (at time *t*) depends only on the previous state of the amino acid $a_{t-1}$ (at time *t−*1): $\text{Pr}(a_{t}|a_{0},a_{1},\ldots ,a_{t-1})=\text{Pr}(a_{t}|a_{t-1}).$ Thus, the conditional probability $\text{Pr}(a_{t}|a_{t-1})$ corresponds to a 1-step transition between states from the time *t−*1.

In this work, M(1) represents a 20 × 20 matrix containing conditional probabilities, where each $M_{ij}$ in the matrix refers to the probability of an amino acid indexed by *j* changing to an amino acid indexed by *i* at *t *=* *1. When choosing the unit of time, we use the convention introduced by Dayhoff *et al.* (1978) and define *t *=* *1 as the discrete time unit taken to observe 1% (= 0.01) *expected* change in amino acids. We shorthand the probability matrix **M** for M(1), and term it the *base matrix*. Each column vector of **M** is an $L_{1}$ normalized vector (i.e. $\sum_{i=1}^{20}M_{ij}=1$ for all 1≤j≤20). Further, as the Markov property holds, M(t) can be computed from M(1) as $M^{t}$, denoting the stochastic matrix after *t* time-steps. Implicit in **M** is its stationary distribution *π* (derived from the eigenvector corresponding to the eigenvalue of 1—the largest eigenvalue of **M**), and $\underset{t\to \infty }{\lim}M^{t}$ gives a matrix whose columns all tend to *π* (Norris and Norris, 1998). Moreover, the eigen-decomposition of **M** enables an efficient computation of $M^{t}$ as $M^{t}=S\Lambda^{t}S^{-1}$, where **S** and Λ are the eigenvector and diagonal eigenvalue matrices of M(1). Note: The above Markov model directly relates to its corresponding continuous-time Markov model via: $M^{t}=e^{Qt},$ where *Q* is an instantaneous rate matrix. It enables a two-way conversion between them given the knowledge of *t* (Kishino *et al.*, 1990; Kosiol and Goldman, 2005). Our framework caters for the inference of the optimal *t* as a discrete variable for all practical purposes. While any **M** stochastic matrix with a valid logarithm carries an implicit rate matrix of its own, a discrete approximation does not obstruct alignment modeling, as we observe that the expected change does not vary significantly within [t,t+1] (see Supplementary Section S3.4).

### 2.4 Multinomial probabilities P to model the amino acids in the indel regions

In general, the multinomial probabilities can be estimated over observations from any finite alphabet $ℵ=\{x_{1},\ldots ,x_{\left|ℵ\right|}\}$ with |ℵ| symbols/states. The MML-estimate for multinomial probabilities (Wallace and Freeman, 1987) has been derived as: $\text{Pr}(x_{i})=\frac{n_{i}+\frac{1}{2}}{\sum_{j=1}^{\left|ℵ\right|}n_{j}+\frac{\left|ℵ\right|}{2}}$, where *n_i_* is the number of observations for each state *x_i_* [refer Allison (2018)]. Using the above, we derive the optimal MML probability estimates **P** by accounting for the number of observations of each amino acid in the indel regions of all alignments in any specified collection **D**.

To provide an alternate estimate for **P**, we compare the above optimal choice with those derived from the stationary distribution *π* of the stochastic matrix **M**. Further, both the above candidates for estimates of **P** are dependent on the observed data **D**. To compare these against an estimate independent of **D**, **P** can be estimated on the UniProt database (Apweiler *et al.*, 2004) (see Supplementary Section S2.2).

### 2.5 Alignment three-state machine and Dirichlet distributions as a function of time

In the collection **D**, any alignment relationship $A_{i}$ for its corresponding sequences $\langle S_{i},T_{i}\rangle$ is described as a 3-state string over the alphabet of {match (m), insert (i), delete (d)} states. This adheres to what was previously considered by Gotoh (1982); Allison *et al.* (1992) for biological sequence alignment, and later by Eddy (1998) with pair hidden Markov models specific to protein sequence alignment.

**Fig. 1.**
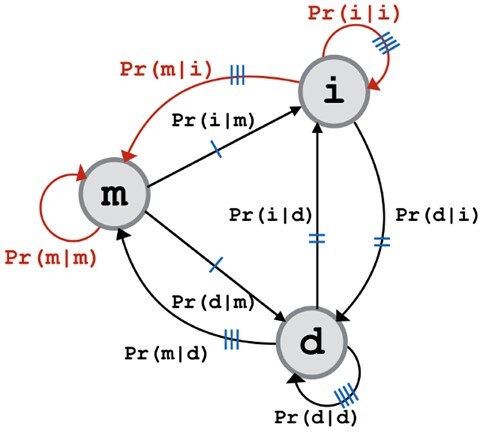
The symmetric three-state machine for modeling an alignment string (Note: the red transitions refer to the three free parameters. Equivalent transitions are indicated in blue.) (A color version of this figure appears in the online version of this article)

This 3-state machine defines nine possible one-step state transitions with corresponding transition probabilities (denoted as Θ) (see Fig. 1), where the sum of probabilities out of any state equals 1. Further, as is common when dealing with alignments of biomolecules, the insert and delete states are treated symmetrically, thus reducing the number of free parameters to three. Notionally these free parameters are represented by {Pr(m|m),Pr(i|i),Pr(m|i)}. The remaining six (dependent) parameters can be derived from them as: $\text{Pr}(i|m)=\text{Pr}(d|m)=\frac{1-\text{Pr}(m|m)}{2}$; Pr(d|i)=Pr(i|d)=1−Pr(i|i)−Pr(m|i); Pr(d|d)=Pr(i|i); Pr(m|d)=Pr(m|i).

These alignment 3-state machine parameters are modeled using Dirichlet distributions inferred for different values of time *t*. In general, a Dirichlet distribution is a natural choice to model the parameters of multistate (categorical) and multinomial distributions, as the Dirichlet is a conjugate prior for them. The literature contains many examples of applying Dirichlet models for other purposes such as amino acid substitution modeling (e.g. Brown *et al.*, 1993; Nguyen *et al.*, 2013).

We define a 1-simplex Dirichlet and a 2-simplex Dirichlet for each discrete time point *t* to model Pr(m|m) and [Pr(i|i),Pr(m|i)], respectively. The time-dependent Dirichlet parameters inferred from **D** are denoted as α(t). See Supplementary Section S1.3.4 for the methodological details of the inference of time-dependent Dirichlet parameters α(t), and Θ given α(t) from any collection of alignments **D**.

### 2.6 Converting a scoring matrix to a stochastic matrix M

Any existing amino acid substitution matrix can be converted into its corresponding stochastic matrix **M** and thus benefit from the MML’s unsupervised parameter estimation. This lends the ability to objectively compare the performance of commonly used substitution matrices, normally published in their linearly scaled *log-odds* form. These log-odds scoring matrices are converted back to their conditional probability form by using their reported multiplier and amino acid frequencies. (Note: Two of the nine matrices we compared—MIQS and PFASUM—do not provide the amino acid frequencies used in their log-odds scores computation, so we used the multinomial probability estimates of amino acids optimal for the collection **D**). Let **C** denote the conditional probability matrix derived from a scoring matrix. Then, **M** is derived from **C** by numerically identifying the *k*th root of the matrix **C**, i.e. $M(1)=C^{\frac{1}{k}}$, such that the resulting **M** is the nearest to having 1% (=0.01) expected amino-acid change (refer Supplementary Section S1.5.2). Once the stochastic matrix is derived from an existing substitution scoring matrix, all the other corresponding parameters (**P**, Θ, α, τ) are automatically inferred using MML, as optimal to **M** for the given collection **D**.

### 2.7 Inference of the best stochastic matrix $M^{*}$ for any benchmark D

This MML framework also allows the inference of a stochastic matrix from an alignment benchmark **D**, the best matrix being the one that minimizes the MML objective function given in Equation (2). To search for the best matrix, we implement a Monte Carlo search method (see Supplementary Section S1.4) that uses simulated annealing (Kirkpatrick *et al.*, 1983). Broadly, beginning from an initial state of **M**, randomly chosen columns of an evolving matrix are perturbed in the near-neighborhood. Using the Metropolis criteria (Metropolis *et al.*, 1953), each perturbation is either accepted/rejected over an iterative Monte Carlo process until convergence (refer Supplementary Section S1.4). Fixing this inferred matrix, we then optimize the 3-state machine parameters described in Section 2.5. We continue to iteratively optimize them until convergence.

### 2.8 Estimation of the terms in Equation (2)

The ‘MML87’ method (Wallace and Freeman, 1987) of parameter estimation is used to compute the Shannon information terms in Equation (2). In general, for a model with continuous parameters *η*, and a prior h(η), Wallace and Freeman (1987) derived the encoding message length required to communicate any observed dataset *d* as I(η,d)=I(η)+I(d|η) bits where, $I(\eta )=-\text{log}[h(\eta )]+\frac{1}{2}\text{log}\;\{\det[\mathrm{Fisher}(\eta )]\}+\frac{\left|\eta \right|}{2}\text{log}(\kappa_{\left|\eta \right|})+\frac{\left|\eta \right|}{2}\;\text{bits}$ [|η| is the number of free parameters, and $\kappa_{\left|\eta \right|}$ is the associated quantizing lattice constant (Conway and Sloane, 1984)], and I(d|η)=L(d) bits where L is the negative log likelihood function, and det(Fisher(η)) is the determinant of the Fisher information matrix (the second derivative of L) that informs the optimal precision required to state *η*. Equation (2) is further described in the Supplementary Section S2.

Finally, we note that the simultaneous inference of the complete set of time-parameterized models, mainly {M(t),α(t)}, given any benchmark alignment is a one-time process, and the complexity of inference is discussed in the Supplementary Section S1.4. Once these models are identified, they can be used to compare any pair of protein sequences in time proportional to the product of lengths of the two sequences using the MML framework of alignment introduced in Sumanaweera *et al.* (2019).

## 3 Results and discussion

Here, we consider nine well-known substitution matrices along with six new matrices derived using our MML framework described in this article. The existing matrices include: PAM (Dayhoff *et al.*, 1978), JTT (Jones *et al.*, 1992), BLOSUM (Henikoff and Henikoff, 1992), JO (Johnson and Overington, 1993), WAG (Whelan and Goldman, 2001), VTML (Müller *et al.*, 2002), LG (Le and Gascuel, 2008), MIQS (Yamada and Tomii, 2014) and PFASUM (Keul *et al.*, 2017). These matrices are compared on six large benchmark collections containing alignments (see Section 2). For each benchmark collection, we further infer a stochastic matrix that best compresses all the alignments in the benchmark. We compare these six MML-inferred matrices against the existing matrices.

In the remainder of this section, the performance of all the matrices across six benchmarks is measured using the Shannon information content. This measure gives the length of lossless compression (in bits) of each benchmark, using respective matrices and their corresponding optimal three-state machine models. From this, one of our six MML-inferred substitution matrices was observed to be the most generalizable matrix across all the benchmarks—we term this matrix, MMLSUM. Further, we detail the characteristics of MMLSUM including aspects of physicochemical and functional properties that the matrix captures, its relationship with the expected change in amino acids as a function of divergence time, and the properties of gaps that can be derived from its companion three-state machine models.

### 3.1 Composition of alignment benchmarks

Table 1 summarizes the six alignment benchmarks. The distributions of sequence identity across these benchmarks are shown in Supplementary Figure SF2. Accordingly, HOMSTRAD covers a wide range of sequence relationships, whereas SABMARK-Sup and MATTBENCH contain alignments of distant sequence-pairs. SABMARK-Twi contains alignments of sequences that have diverged into the ‘midnight zone’ (Rost, 1999) where the detectable sequence signal is extremely feeble. Finally, the largest benchmark contains 59 092 unique sequence-pairs sampled from the superfamily and family levels of SCOP (Murzin *et al.*, 1995). These pairs were aligned separately using DALI (Holm and Sander, 1993) and MMLigner (Collier *et al.*, 2017) structural alignment programs to obtain SCOP1 and SCOP2 benchmarks, respectively (Note: of the 59 092 pairs in the sampled SCOP data-set, DALI does not report any alignment for 2800 pairs). Collectively, all six benchmarks cover varying distributions of sequence relationships. This diversity of chosen benchmarks minimizes the possibility of introducing any systematic bias to the evaluation.

**Table 1.** Composition of the structural alignment benchmarks used in this work

Benchmark						
Name	Curated with structural aligner	No. of aligned pairs	No. of matches	No. of inserts	No. of deletes	Average sequence identity
HOMSTRAD (Mizuguchi *et al.*, 1998)	MNYFIT, STAMP, COMPARER	8323	1 311 478	96 911	98 810	35.1%
MATTBENCH (Daniels *et al.*, 2012)	MATT	5286	826 506	177 401	177 789	19.4%
SABMARK-Sup (Van Walle *et al.*, 2005)	SOFI, CE	19 092	1 750 440	848 859	861 344	15.2%
SABMARK-Twi (Van Walle *et al.*, 2005)	SOFI, CE	10 667	694 954	515 318	527 188	8.4%
SCOP1 (Andreeva *et al.*, 2020)	DALI	56 292	8 663 652	1 407 988	1 373 882	25.5%
SCOP2 (Andreeva *et al.*, 2020)	MMLIGNER	59 092	8 145 678	1 673 687	1 653 531	24.8%

### 3.2 Measuring Shannon information content of benchmarks using different substitution matrices

We estimated the lossless encoding message length for each of the above benchmarks using our MML framework described in Section 2. The framework quantifies the Shannon information content of a benchmark, measured in *bits*, under each of the substitution models.

Further, following the notations described in Section 2, for a stochastic matrix **M** chosen to compress losslessly all the sequence-pairs in a specific alignment benchmark D, all the other models involved in this MML information-theoretic framework, i.e. {P,α,Θ,τ} (denoting the 20-nomial model of amino acids, Dirichlet distribution parameters, alignment three-state machine parameters and evolutionary time parameters, respectively), are automatically inferred (optimized) for **M** on each benchmark D under the MML criterion [Note: In the context of model evaluation using Shannon information, the lossless compression of data is expected to reach the minimum (optimal) encoding length when the underlying probabilistic model approaches the (unknown) true model. Hence, a lower encoding length is indicative of better models].

Table 2 presents the lengths of the shortest encoding (i.e. Shannon information) using each of the 15 matrices (9 existing; 6 inferred) to explain each benchmark.

**Table 2.** Shannon information content (in bits) to ecnode losslessly each structural alignment benchmark by varying the substitution matrices

Benchmark →	HOMSTRAD	MATTBENCH	SABMARK-Sup	SABMARK-Twi	SCOP1	SCOP2
Matrix (*M*) ↓	**Shannon information content using existing substitution matrices (and its rank across all matrices**)					

PAM (1978)	11531556.4 (15)	9143136.9 (15)	23574085.5 (15)	11310226.0 (15)	84925406.9 (15)	82757945.5 (15)
JTT (1992)	11481203.2 (14)	9072068.6 (13)	23450831.6 (13)	11251914.3 (13)	84353986.5 (13)	82218532.0 (13)
BLOSUM (1992)	11437552.8 (10)	9037049.8 (08)	23373908.1 (07)	11228043.6 (06)	84174710.8 (11)	81995179.3 (10)
JO (1993)	11476518.5 (13)	9118266.0 (14)	23501361.5 (14)	11290056.3 (14)	84567477.8 (14)	82405562.0 (14)
WAG (2001)	11419186.0 (05)	9052722.9 (12)	23400017.0 (11)	11243242.0 (12)	84141633.8 (09)	81996154.3 (11)
VTML (2002)	11423498.2 (07)	9035903.4 (07)	23377505.0 (08)	11230624.1 (08)	84075908.5 (07)	81925302.7 (07)
LG (2008)	11464263.6 (12)	9049040.6 (11)	23411713.3 (12)	11235389.2 (09)	84255656.9 (08)	82090289.0 (12)
MIQS (2013)	11422215.4 (06)	9040480.8 (10)	23385242.8 (10)	11236323.3 (10)	84076742.8 (12)	81927707.6 (08)
PFASUM (2017)	11412888.2 (02)	9039799.4 (09)	23379074.4 (09)	11236572.3 (11)	84040519.3 (04)	81902713.6 (06)

Matrix (*M*) ↓	**Shannon information using MML-inferred matrices (and its rank across all matrices**)					

${\text{MML}}_{\mathrm{HOMSTRAD}}$	**11405604.7 (01)**	9035317.6 (05)	23365151.2 (06)	11230184.9 (07)	84026302.3 (03)	81873575.9 (03)
${\text{MML}}_{\mathrm{MATTBENCH}}$	11426344.1 (09)	**9025882.4 (01)**	23355215.8 (03)	11217219.1 (03)	84050927.4 (05)	81886796.3 (04)
${\text{MML}}_{\mathrm{SABMARK-Sup}}$	11424135.9 (08)	9031315.9 (04)	**23346025.4 (01)**	11212252.7 (02)	84067892.9 (06)	81889152.3 (05)
${\text{MML}}_{\mathrm{SABMARK-Twi}}$	11442781.7 (11)	9035720.5 (06)	23356054.5 (05)	**11211360.9 (01)**	84155701.5 (10)	81962307.9 (09)
${\text{MML}}_{\mathrm{SCOP1}}$	11413295.6 (03)	9029682.8 (03)	23355235.9 (04)	11221295.6 (05)	**83996796.0 (01)**	81848381.0 (02)
${\text{MML}}_{\mathrm{SCOP2}}$ (MMLSUM)	11413725.3 (04)	9028667.52 (02)	23349826.8 (02)	11218205.6 (04)	83999536.4 (02)	**81840654.6 (01)**

*Note*: The rank order of their performance is reported in each column within parentheses (smaller lossless encoding length = better rank). Best encoding length for each benchmark is highlighted in bold. All other parameters are inferred (optimized) by the MML framework for the matrix chosen to compress losslessly each benchmark.

Previously published matrices are arranged in their chronological order of publication (rows). The last five rows show results for the stochastic matrices inferred from each benchmark. To get an overall view of the performance of each matrix as a consensus over all benchmarks generated from individual ranks (shown within parentheses in each column of Table 2), we use a simple yet effective statistic: the (row-wise) sum of ranks of each matrix over all benchmarks, ranksum in short. Since this evaluation involves the ranking of 15 matrices over 6 benchmarks, the ranksum of any matrix is an integer between 6 × 1 = 6 (best possible performance) and 6 × 15 = 90 (worst possible performance).

Amongst the existing matrices, PAM (ranksum = 90) consistently gave the worst (i.e. longest) lossless encoding lengths across all benchmarks. This is anticipated, as PAM was derived in 1970s using the then available (limited) set of closely related protein relationships. This is improved upon by JO (ranksum = 83), JTT (ranksum = 79), LG (ranksum = 64), WAG (ranksum = 60), MIQS (ranksum = 56), BLOSUM (ranksum = 52), VTML (ranksum = 44) and PFASUM (ranksum = 41). These numbers suggest that, by and large, the previously published amino acid substitution models have improved over time, becoming more representative of the current protein corpus. BLOSUM (1992) is amongst the earliest matrices to outperform several matrices that were proposed much later, and is only superseded in performance by VTML (2002) and PFASUM (2017) amongst the recent matrices.

Further, we observe that the MML-inferred stochastic matrices, specific to each benchmark, perform consistently better than previously published substitution matrices. Indeed, it is to be *expected* that the encoding length of any MML-inferred matrix optimized on a specific benchmark will outperform all the other matrices on that particular benchmark—and this is precisely what is observed in Table 2. However, the utility of any matrix lies in its ability to generalize to *other* benchmarks and perform well on those. The results clearly demonstrate the ability of MML-inferred matrices to generalize and explain other benchmarks, far outperforming all existing ones. According to their ranksums: MML${}_{\mathrm{SABMARK-Sup}}$ (i.e. the stochastic matrix inferred from SABMARK-Sup) gives ranksum = 26 across all benchmarks, while MML${}_{\mathrm{MATTBENCH}}$ and MML${}_{\mathrm{HOMSTRAD}}$ give ranksum = 25. The top two performers overall come from the matrices inferred on the two SCOP benchmarks, MML${}_{\mathrm{SCOP1}}$ (ranksum = 18) and MML${}_{\text{SCOP}2}$ (ranksum = 15). The sole outlier amongst the MML-inferred matrices is MML${}_{\text{SABMARK-Twi}}$ with ranksum = 42. As already stated, SABMARK-Twi benchmark contains alignments of highly diverged sequence-pairs (with an average sequence identity of 8.4%). Thus, the benchmark itself carries an extremely weak sequence signal, deflecting the inference of a stochastic matrix that can generalize effectively to explain a wider range of sequence relationships that other benchmarks embody. Nevertheless, a noteworthy observation is that MML${}_{\mathrm{SABMARK-Twi}}$ (ranksum = 42) is nearly in par with PFASUM (ranksum = 41) which was the best performer amongst the set of existing matrices.

Supplementary Sections S2–S3 present an extended analysis and additional information for this comparative study. Overall, the MML-inferred matrix from the SCOP2 benchmark (${\text{MML}}_{\mathrm{SCOP2}}$) with a ranksum = 15 outperforms all other matrices. This is because the SCOP2 benchmark is three times larger than SABMARK-Sup (seven times that of HOMSTRAD) and contains a wider range of sequence relationships than the other benchmarks. Thus, all of our subsequent analyses will involve the ${\text{MML}}_{\mathrm{SCOP2}}$ matrix—we will refer to this as MMLSUM.

### 3.3 Analysis of MMLSUM (MML SUbstitution Matrix)

MMLSUM above is shown to be effective in compressing all alignment benchmarks compared to the other existing substitution matrices, reflecting a reliable representative of average amino acid substitution patterns observed across the present repertoire of proteins. We now analyze the protein physicochemical and functional properties that it captures, the expected amino acid change as a function of divergence time, and the properties of gaps derived from its companion probabilistic models.

#### 3.3.1 Amino acid clusters

An amino acid substitution matrix encompasses average similarities and differences present amongst possible substitution patterns, reflecting physicochemically similar groups of amino acids. Checking amino acid clusters implicit in a substitution matrix has been historically flagged as a viable method of checking if the particular matrix captures sensible patterns of substitutions (Dayhoff *et al.*, 1978; French and Robson, 1983). Here, we analyze amino acid groups implicit in MMLSUM, using (i) hierarchical clustering, and (ii) tSNE embedding.

**Fig. 2.**
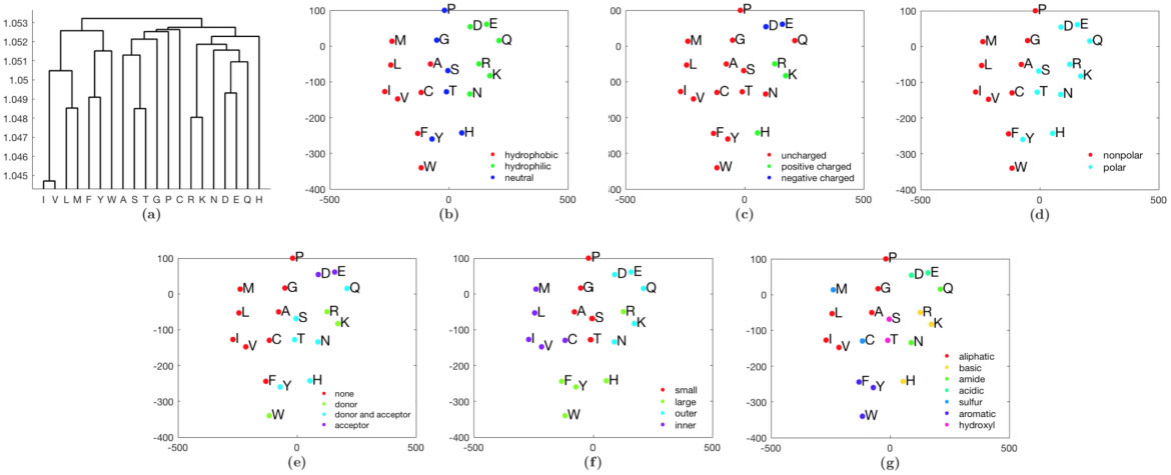
(**a**) Average-linkage clustering of amino acids generated from MMLSUM. (**b**–**g**) tSNE clustering of amino acids generated from MMLSUM. All plots have the same clustering, but colored under different amino acid classification schemes based on: (b) hydropathy, (c) charge, (d) polarity, (e) hydrogen donor or acceptor role, (f) volume and exposure (Swanson, 1984) and (g) chemical properties [based on IMGT classification (Lefranc *et al.*, 2015) and Swanson classification of amino acids (Swanson, 1984)]. Refer to the legend for the coloring scheme in various subplots

Figure 2a gives a dendrogram generated using the average-linkage method on the MMLSUM base stochastic matrix (representing evolutionary time *t *=* *1, denoting 1% expected change in amino acids). We notice the following important clusters of amino acids, previously marked as necessary for a reliable matrix to capture (Dayhoff *et al.*, 1978): (i) Hydrophobic amino acids—[Valine (V), Isoleucine (I), Leucine (L), Methionine (M)]; (ii) Aromatic amino acids—[Tryptophan (W), Tyrosine (Y), Phenylalanine (F)]; (iii) Neutral amino acids—[Alanine (A), Serine (S), Threonine (T), Glycine (G), Proline (P)]; (iv) Large amino acids—[Arginine (R), Lysine (K), Asparagine (N), Aspartic acid (D), Glutamic acid (E), Glutamine (Q)]; The remaining two amino acids, Histidine (H) and Cysteine (C) cluster apart from the rest. To study the groupings more systematically and from a different point of view, we apply the non-linear dimensionality reduction technique of t-distributed stochastic neighbor embedding (tSNE) (van der Maaten and Hinton, 2008) to MMLSUM. Figure 2b–g all shows the same 2D tSNE plots of MMLSUM, but each subplot colors the amino acids differently based on widely used classification schemes. These schemes encompass the hydropathic character, charge, polarity, donor/acceptor roles in forming hydrogen bonds, size and propensity for being buried/exposed and the chemical constitution of amino acids. In Figure 2b–f, the tSNE yields clearly separable amino acid groups on the 2D embedding of MMLSUM. In Figure 2g where the classification is based on the chemical characteristics of amino acids [as per IMGT (Lefranc *et al.*, 2015)], the classes are mostly well-differentiated, barring a few outliers that include Histidine (H), Cysteine (C) and Asparagine (N)—we note that H and C were also outliers in the hierarchical clustering (cf. Fig. 2a).

#### 3.3.2 Expected amino acid change as a function of divergence time

**Fig. 3.**
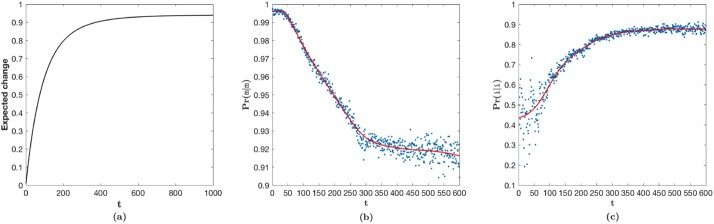
(**a**) Amino acid expected change under MMLSUM as a function of divergence-time *t*. (**b**) The variation of Pr(m|m) when derived from the *mean* of the inferred time-dependent Dirichlet distributions accompanying MMLSUM. (**c**) Similarly, the variation of Pr(i|i) estimate with *t*. Note: For (b) and (c), *t* is plotted in the range [1,600] beyond which the amino acids have near-converged to the stationary distribution (see Supplementary Fig. SF6)

The expected change of a stochastic matrix is defined as the probability of observing a change in any state (on average)—see Supplementary Section S1.5.1 for more details. Figure 3a shows the growth of expected change of amino acids implicit in MMLSUM as a function of divergence ‘time’ *t*. Previous studies (Rost, 1999) have shown that protein sequence relationships are most reliable when their sequence identity is >40% (or the expected amino acid change is <60%). This corresponds approximately to the evolutionary time t∈[1,100] of MMLSUM. The ‘twilight zone’ of sequence relationships has been characterized by relationships sharing [20−35]% sequence identity (or [65−80]% change). This corresponds approximately to the range t∈[125−200] of MMLSUM. Expected change of 90% is reached at *t *=* *400, which then increases very slowly thereafter (∼94% change at *t *=* *1000) (see Supplementary Section S3.3 for an extended analysis).

#### 3.3.3 Matched block and gap lengths as a function of divergence time

Significant to this framework is the unified treatment of substitutions and gaps via optimal ‘time’(*t*)-parameterized Dirichlet distributions. These Dirichlet models enable the analysis of expected matched block lengths and gap lengths in protein alignments, as a function of *t*. They model time-specific state transition probabilities of the alignment three-state machine over match (m), insert (i) and delete (d) states (see Fig. 1). Section 2 introduces the 9 transition probabilities involved in the alignment three-state machine, of which three are free (i.e. Pr(m|m), Pr(m|i) and Pr(i|i)) and the remaining are dependent. Note that, the three-state machine implicitly carries the notion of the widely used affine gap function which supports biologically realistic *en bloc* indels and computational efficiency (Cartwright, 2006; Gotoh, 1982; Rivas and Eddy, 2015). There, a match block length or a gap length is modeled as a random variable over a geometric distribution.

In the three-state machine, the probability of moving from a match state to another match state (Pr(m|m)) controls the run length of any block of matches in an alignment. The expected value of this run length, a geometrically distributed variable, is given by $\frac{1}{1-\text{Pr}(m|m)}$. Also, the value 1−Pr(m|m) gives the probability of a gap (i.e. a block of insertions or deletions of any length) starting at a given position in an alignment. Figure 3b plots the values of Pr(m|m) derived from the mean values of the inferred Dirichlets for the match state. We observe that it remains nearly a constant (Pr(m|m) = 0.9958 on average) in the range of t∈[1,40]. This value corresponds to an *expected* run length of ∼238 amino acids per block of matches. Sequence-pairs whose time parameter is in that range are closely related, with >67% amino acids expected to be *conserved* (cf. Fig. 3a). The probability of opening a gap (1−Pr(m|m)=0.0042) for sequence-pairs in this range is extremely small. Next in the range t∈[40,300], Pr(m|m) decreases linearly with *t*. Comparing this range for the expected change of amino acids (Fig. 3a), we can see that it drastically increases from ∼32% to ∼87%. This correlates with the expected length of match-blocks dropping from 238 amino acids to about 13. Further, for t≥300, Pr(m|m) decreases only gradually.

Similarly, the free parameter Pr(i|i) (equivalent to Pr(d|d) in the *symmetric* alignment state machine) controls the run lengths of indels. Figure 3c gives values of Pr(i|i) derived from the mean values of the inferred Dirichlets for the i state. In the range t∈[1,50], values of Pr(i|i) are noisy because the probability of a gap is small. Hence, there are only a few observed gaps from which to estimate this parameter. However, in the range of t∈[50,400], Pr(i|i) grows from 0.5248 to about 0.8431 beyond which the probability flattens out at about 0.8759 on average (expected gap length $\frac{1}{1-\text{Pr}(i|i)}=∼8$ amino acid residues). The change of Pr(m|i) with *t* mirrors the behavior of Pr(i|i), as: Pr(i|i)+Pr(m|i)+Pr(d|i)=1 and Pr(d|i) remains very small.

#### 3.3.4 Similarity of protein function versus the time of divergence

The following is a complementary analysis on how function similarity between protein domain-pairs in the largest representative benchmark of our selection (i.e. SCOP2) correlates with the estimated time (of divergence) parameter under MMLSUM [refer Supplementary Fig. SF5 for the distribution of the inferred set of time parameters]. For this, we use the Gene Ontology (GO) resource (http://geneontology.org/) that provides function annotations for protein domains in three categories: (i) the ‘Biological Process’ (BP) they come from, (ii) the ‘Molecular Function’ (MF) they exhibit and (iii) the ‘Cellular Component’ (CC) they belong to. Due to missing tags in the GO database, not all 59 092 pairs in SCOP2 could be considered for the analysis presented here: we considered only those domain-pairs where both domains have one or more of the above categories tagged in the GO database. This resulted in 37 201 pairs at the level of BP, 48 215 pairs at the level of MF and 31 594 at the level of CC, for the exploration of function similarity.

**Fig. 4.**
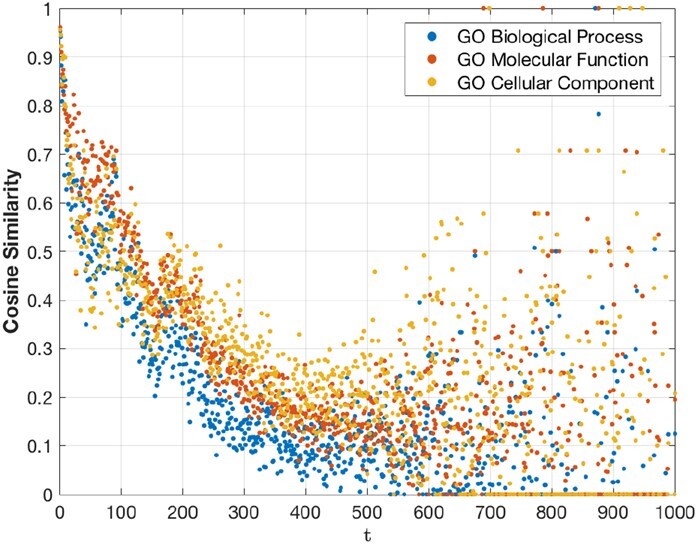
Similarity of the GO annotations of two aligned proteins varies as a function of their evolutionary divergence *t* under MMLSUM

The function similarity between a domain pair is evaluated using a similarity measure involving their list of terms within each GO category as follows. Each domain is represented as a Boolean vector corresponding to the observed set of distinct terms in the GO database. For each domain-pair, two such vectors $\vec{x}$ and $\vec{y}$ are constructed and their cosine similarity $\frac{\vec{x}\cdot \vec{y}}{\left||\vec{x}||||\vec{y}|\right|}$ is computed. Figure 4 plots the average changes of this measure as a function of *t*. Overall, the observed trend seen in Figure 4 is consistent with the plot showing the convergence of amino acids to the stationary distribution of MMLSUM (cf. Supplementary Fig. SF6). As expected, the function-similarity measure decreases as the domains diverge from each other and thereby pick up new functions. The similarity measure flattens out (and becomes noisy) for *t *>* *400.

Interestingly, studying the ‘phylum’ of each domain reveals the divergence of function from another point of view. We analyzed the proportion of SCOP2 domain-pairs where both domains belong to the same phylum, by binning their inferred time parameters using MMLSUM. We find that 92.3% of the domain-pairs whose inferred time parameters are in the range t∈[1,50] belong to the same phylum. Between t∈(50,100] this proportion falls to 53.5%. We observe a roughly similar proportion of 50.4% for the values of t∈(100,200]. Between t∈(200,300] and t∈(300,600], the number drops more drastically to 34.1% and 32%.

Finally, Supplementary Section S3.5 discusses a case study involving a diverging set of 9 Globin sequences, in order to explore the ability of the inferred models here to detect sequence relationships (and their correlation with inferred Markov time) across varying evolutionary distances of their host species.

### 3.4 Conclusions

We combined a time-dependent substitution matrix and an associated three-state alignment machine in a Bayesian and information-theoretic framework, to give a unified statistical model of aligned protein sequences This model provides many advantages, including the inference of evolutionary divergence time between two proteins, the Shannon information estimation of any benchmark containing protein alignments, an objective comparison of widely used substitution matrices, and the inference of a newly improved Markov model of protein evolution. The framework was developed using the statistical inference criterion of Minimum Message Length (MML) (Wallace and Freeman, 1987).

Here, we outlined the significant improvement of substitution modeling gained over the past forty years of history by comparing nine popular substitution matrices. The study accommodated both, Markov and non-Markov models of protein evolution, by converting matrices which do not explicitly depend on time to corresponding stochastic matrices that *can* benefit from our information-theoretic framework. We also inferred optimal Markov matrices for each of six structural alignment benchmarks. This allowed us to compare the nine existing matrices with the six MML-inferred matrices on the six benchmarks. All MML-inferred matrices perform very well. In particular, the MMLSUM matrix outperforms other matrices and generalizes best across the set of benchmarks. MMLSUM also implies sensible groupings of the 20 amino acids. Moreover, the complete statistical model yields an interesting relationship between evolutionary time and the frequency and length of gaps (indels). Furthermore, the inferred evolutionary time correlates well with protein function similarity. Overall, the new models and insights resulting from this study will be useful to advance the field of protein alignment (e.g. to evaluate new matrices, infer new matrices). All models compared and inferred in this study (along with the programs and raw data) is downloadable from https://lcb.infotech.monash.edu.au/mmlsum.

User runs using any of the above models to compare pairs of protein sequences and reason their relationship can be carried out using the latest version of seqMMLigner (Sumanaweera *et al.*, 2019), freely downloadable from https://lcb.infotech.monash.edu.au/seqmmligner. Finally, MMLSUM matrices introduced in this work have been used to estimate the reliability and establish the limits of inference of alignment relationships from amino acid sequence information (Rajapaksa *et al.*, 2022).

## Funding

D.S.’s PhD was funded by Monash University. A.K.’s research was funded by Australian Research Council’s Discovery Project [DP150100894].

*Conflict of Interest*: none declared.

## Supplementary Material

btac246_Supplementary_Data
