# Supplementary material for: Bridging the gaps in statistical models of protein alignment
Source: Bioinformatics. 2022 Jun 27;38(Suppl 1):i229–37. doi: 10.1093/bioinformatics/btac246 (PMC9235498; doi:10.1093/bioinformatics/btac246)
Supplement: btac246_Supplementary_Data [file btac246_supplementary_data.pdf]

Supplementary notes for "*Bridging the Gaps in Statistical Models of Protein Alignment*"

Dinithi Sumanaweera, Lloyd Allison* and Arun S. Konagurthu*
Department of Data Science and Artificial Intelligence, Faculty of IT, Monash University, Australia

S1 Supplementary Methods

S1.1 Overview to the inference methodology

As described in the **Methods and Material** of the main text, given any benchmark **D** containing a collection of alignments (as 3-state strings) and their associated protein sequences, our framework involves simultaneous inference of the following statistical models:

- the stochastic matrix **M** used to explain the matched pairs of amino acids observed in **D**;
- the multinomial probability estimates **P** to explain the amino acids in the indel regions in **D**;
- the divergence time parameter $t_i \in \boldsymbol{\tau}$, one for each alignment in **D**;
- the 3-state transition probabilities $\boldsymbol{\Theta}_i$ for each alignment 3-state string in **D**; and
- the "time"-dependent Dirichlet distributions $\boldsymbol{\alpha}$ modelling the 3-state probabilities of all alignments in **D**.

Of the above, the inference of the optimal MML estimates of multinomial probabilities **P** is fully independent of the inference of other models. These MML estimates are dependent solely on the insertions and deletions observed in the benchmark **D**. However, the remaining parameters $\mathbf{M}, \boldsymbol{\alpha}, \boldsymbol{\Theta}$, and $\boldsymbol{\tau}$ have to be inferred simultaneously. Therefore, we rely on an iterative approach similar to an Expectation-Maximisation (EM) strategy. Under a monotonically decreasing value of the objective function (in this case the lossless encoding length), a set of parameters are held fixed (specifically $\boldsymbol{\alpha}$ and $\boldsymbol{\Theta}$) to optimise for the remaining parameters (specifically, **M** and $\boldsymbol{\tau}$). Then these (**M** and $\boldsymbol{\tau}$) are held fixed, to optimise the former ($\boldsymbol{\alpha}$ and $\boldsymbol{\Theta}$). This process is repeated until the total lossless encoding length does not change between successive iterations.

Broadly, the method of Wallace and Freeman [34] (referred to as the MML87 method) lays the foundation on which model-parameters are inferred, and respective encoding lengths (given in Eq. 2 of the main text) are estimated. As stated in the main text **Methods and Material** section, for any model with continuous parameters η, and prior $h(\eta)$, Wallace and Freeman [34] derived the following message length required to explain any observed data-set d: $I(\eta, d) = I(\eta) + I(d|\eta)$ with

$$I(\eta) = -\log\left[h(\eta)\right] + \frac{1}{2}\log\left\{det[Fisher(\eta)]\right\} + \frac{|\eta|}{2}\log\left(c_{|\eta|}\right) + \frac{|\eta|}{2} \quad (1)$$

$$I(d|\eta) = \mathcal{L}(d) \quad (2)$$

where $|\eta|$ is the number of free parameters; $c_{|\eta|}$ is the associated lattice constant [8]; and $det(Fisher(\eta))$ is the determinant of the *expected* Fisher information matrix (the expected value of the second derivative of the negative log likelihood function) that informs the optimal precision required to state η. In MML, only the free parameters require encoding (since all dependent parameters can be deduced from the free ones).

Below, we will first discuss the estimation of encoding lengths of various terms involved in Eq. 2 in the main text, followed by the details of the search methods for optimising the aforementioned model-parameters.

S1.2 Introduction to Dirichlet distributions

Dirichlet distributions are used here to losslessly encode (using MML) the transition probabilities of a 3-state machine. In general, a Dirichlet probability distribution models d-dimensional points in the standard unit $(d-1)$-simplex [1]. We denote a Dirichlet distribution as $\text{Dir}(\vec{\alpha})$, with a parameter vector $\vec{\alpha} = [\alpha_1, \alpha_2, \ldots,$

*Corresponding Author

$\alpha_d]$. This models the distribution of a random variable $\vec{\Theta} = [\theta_1,\ \theta_2,\ \dots,\ \theta_d]$ (such that $\sum_{i=1}^{\mathrm{d}} \theta_i = 1$) whose values represent a d-dimensional probability vector. We note that $\vec{\Theta}$ has $d-1$ degrees of freedom, while $\vec{\alpha}$ has d degrees of freedom.

Dirichlet distributions are best understood using the following reparameterisation of $\vec{\alpha}$:

$$\vec{\alpha} = \kappa \cdot \hat{\mu} = \underbrace{\sum_{i=1}^{d} \alpha_i}_{\kappa} \cdot \underbrace{\left[\frac{\alpha_1}{\sum_{i=1}^{d} \alpha_i},\ \frac{\alpha_2}{\sum_{i=1}^{d} \alpha_i}, \dots,\ \frac{\alpha_d}{\sum_{i=1}^{d} \alpha_i} \right]}_{\hat{\mu}}$$

where the distribution's spread is controlled by the concentration parameter κ defined about the distribution's mean $\mathbb{L}1$-normalized vector $\hat{\mu}$. In this work, all "time"-dependent $\vec{\alpha}$ are inferred in terms of their respective κ and $\hat{\mu}$ values.

A Dirichlet probability density function is denoted as:

$$\mathrm{Dir}(\vec{\Theta}; \vec{\alpha}) = \frac{1}{\mathrm{B}(\vec{\alpha})} \prod_{i=1}^{\mathrm{d}} {\theta_i}^{\alpha_i - 1} \tag{3}$$

where $\mathrm{B}(\vec{\alpha}) = \dfrac{\prod_{i=1}^{d} \Gamma(\alpha_i)}{\Gamma(\kappa)}$ is the multivariate Beta function defined using the Gamma function ($\Gamma(.)$)

Computing the Fisher information of Dirichlet distribution. The Fisher information matrix of Dirichlet distribution is given by the second derivative of its negative log likelihood function. The likelihood over data $\mathbf{\Theta}$ containing N samples $\mathbf{\Theta} = \{\vec{\Theta}_1, \dots, \vec{\Theta}_N\}$ (where each $\vec{\Theta}_j, \forall 1 \le j \le N$, is of the form $[\theta_{j_1}, \dots, \theta_{j_d}]$), is defined by the function $f(\mathbf{\Theta}|\vec{\alpha}) = \prod_{j=1}^{\mathrm{N}} \mathrm{Dir}(\vec{\Theta}_j;\vec{\alpha})$; the negative log likelihood function is of the form:

$$\mathcal{L}(\mathbf{\Theta}|\vec{\alpha}) = -\mathrm{N} \log \Gamma(\kappa) + \mathrm{N} \sum_{j=1}^{\mathrm{d}} \log \Gamma(\alpha_j) - \sum_{i=1}^{\mathrm{N}} \sum_{j=1}^{\mathrm{d}} (\alpha_j - 1) \log (\theta_{j_i})$$

The matrix containing second partial derivatives of the above function with respect to the Dirichlet parameters $\vec{\alpha}$ gives the Fisher information matrix, whose derivative is (refer [1, section 2.3])

$$det[Fisher(\vec{\alpha})] = \mathrm{N}^{\mathrm{d}} \{ \prod_{i=1}^{\mathrm{d}} \psi_1(\alpha_i) \} \{ 1 - \psi_1(\kappa) (\sum_{i=1}^{\mathrm{d}} \frac{1}{\psi_1(\alpha_i)}) \} \tag{4}$$

where $\psi_1(\cdot)$ is the polygamma function of order 1 (a.k.a. trigamma function). Note, the determinant of the associated Fisher matrix, $Fisher(\vec{\alpha})$, indicates how sensitive the expected negative log likelihood function $\mathcal{L}$ is to the changes of $\vec{\alpha}$. This determinant is among the terms that dictates the optimum precision to which continuous parameters (in this case $\vec{\alpha}$) need to be stated, and influences their optimal encoding length (see Eq. 1).

Sampling a d-dimensional probability vector from a given Dirichlet distribution. A d-dimensional probability vector $\vec{\Theta} = (\theta_1, ..., \theta_d)$ can be sampled from a d-dimensional $\mathrm{Dir}(\vec{\alpha})$ as follows:

1. Each component θ_i is treated as a Gamma distributed sample y_i generated from Gamma distribution $\mathrm{Gamma}(\alpha_i, 1)$.
2. Repeating the above d times, we get a vector $\vec{y} = (y_1, \dots, y_d)$
3. $\vec{y}$ is $\mathbb{L}_1$-normalized to generate $\vec{\Theta}$.

The resultant vector $\vec{\Theta}$ with $\theta_i = \frac{y_i}{\sum_{i=1}^{d} y_i}$ is Dirichlet distributed under $\mathrm{Dir}(\vec{\alpha})$.

Message length of stating a probability vector $\vec{\Theta}$ using a Dirichlet model $\mathrm{Dir}(\vec{\alpha})$**.** In general, let $\vec{\Theta} = (\theta_1, ..., \theta_d)$ denote a d-dimensional probability vector, where each θ_i denotes the probability of each distinct state x_i, whereas $count(x_i)$ is the number of times x_i is observed. This implies, $\vec{\Theta}$ has $(d-1)$ degrees of freedom (notionally, say $\theta_d = 1 - \sum_{i=1}^{d-1} \theta_i$). Since, $\sum_{i=1}^{d} \theta_i = 1$, $\vec{\Theta}$ is a point inside a $(d-1)$-simplex, modelled using a Dirichlet distribution $\mathrm{Dir}(\vec{\alpha})$.

Using the MML method of Wallace and Freeman [34], the statement cost of $\vec{\alpha}$ and $\vec{\Theta}$ given $\vec{\alpha}$ (denoted by $\vec{\Theta}|\vec{\alpha}$) is given by: $I(\vec{\alpha}, \vec{\Theta}) = I(\vec{\alpha}) + I(\vec{\Theta}|\vec{\alpha})$ where,

$$I(\vec{\alpha}) \quad = \quad -\log [h(\vec{\alpha})] + \frac{1}{2} \log (det[Fisher(\vec{\alpha})]) + \frac{d}{2} \log (c_d) + \frac{d}{2} \tag{5}$$

$$I(\vec{\Theta}|\vec{\alpha}) \quad = \quad -\log [\mathrm{Dir}(\vec{\Theta}_i; \vec{\alpha})] + \frac{1}{2} \log (det[Fisher(\vec{\Theta}_i)]) + \frac{d-1}{2} \log (c_{d-1}) + \frac{d-1}{2} \tag{6}$$

In Equations 5-6, $h(\vec{\alpha})$ is any specified prior on Dirichlet parameters $\vec{\alpha}$, whereas $\text{Dir}(\vec{\alpha})$ is the Dirichlet prior on $\vec{\Theta}$. c_d and c_{d-1} are the optimal lattice constants [8] associated with d degrees of freedom in $\vec{\alpha}$ and $(d-1)$ degrees of freedom in $\vec{\Theta}$, respectively. Further, $det[Fisher(\vec{\alpha})]$ is the determinant of the expected Fisher information of $\vec{\alpha}$ given in Equation 4, that informs the optimal precision to which that parameter should be stated in MML. Similarly, $det[Fisher(\vec{\Theta})]$ given in Equation 7 below is the expected Fisher information of $\vec{\Theta}$ that informs the optimal precision to which $\vec{\Theta}$ is stated, as computed by MML [4, 34].

$$det[Fisher(\vec{\Theta})] = \frac{\left(\sum_{i=1}^{d} count(x_i)\right)^{d-1}}{\theta_i \theta_2 \cdots \theta_d} \tag{7}$$

Note, $\sum_{i=1}^{d} count(x_i)$ is the total number of observations (combining all states).

S1.3 Computing the terms in Eq. 2 of the main text

S1.3.1 Computation of $I(\mathbf{M})$

A stochastic matrix $\mathbf{M}$ is a 20×20 matrix where the value in any cell M_{ij} is the conditional probability of an amino acid indexed by j changing to an amino acid indexed by i, in one unit of time. Since $\sum_{i=1}^{20} \mathbf{M}_{ij} = 1$, each column M_j is a $(d = 20)$-dimensional $\mathbb{L}_1$-normalized multinomial probability vector (over the amino acid states). Therefore, the statement cost of the whole matrix $\mathbf{M}$ can be described as the sum of individual statement costs associated with each of its 20 columns:

$$I(\mathbf{M}) = \sum_{j=1}^{20} I(\mathbf{M}_j)$$

Using a uniform prior on each $\mathbf{M}_j$ (which implies a Dirichlet distribution with $\vec{\alpha} = \{1, 1, \ldots, 1\}$), the statement lengths $I(\mathbf{M}_j), \forall 1 \leq j \leq 20$ can be computed using Equation 6.

S1.3.2 Computation of $I(\mathbf{P})$

In general, the MML87 estimate $\theta_i \in \vec{\Theta}$ for any state x_i that is observed $counts(x_i)$ times, is derived as:

$$\theta_i = \frac{counts(x_i) + \alpha_i - \frac{1}{2}}{\sum_{i=1}^{d}(counts(x_i) + \alpha_i) - \frac{d}{2}} \tag{8}$$

Here, $\mathbf{P}$, that is used in this framework to encode amino acids in the indel regions of alignments in a given benchmark $\mathbf{D}$, is therefore inferred as a $(d = 20)$-nomial probability vector containing the probability estimates of all distinct amino acids, as observed in the indel regions. Thus, the 20-dimensional $\mathbf{P}$ vector can be inferred from observations of indels in $\mathbf{D}$, as per Equation 8.

Further, under the assumption that the prior on $\mathbf{P}$ is uniform (i.e. $\vec{\alpha} = \{1, 1, \ldots, 1\}$), the statement length $I(\mathbf{P})$ can be computed again using Equation 6.

S1.3.3 Estimating time $t_i \in \boldsymbol{\tau}$ of any alignment $\mathcal{A}_i \in \mathbf{D}$ and the computation of $I(t_i)$

For a given set of parameters $\mathbf{M}, \mathbf{P}, \boldsymbol{\alpha}, \boldsymbol{\Theta}$ (i.e. when they are held fixed within any iteration of our EM-like approach – refer Section S1.1), these parameter values allow the estimation of an optimal time parameter $t_i \in \boldsymbol{\tau}$ (modelling the divergence of the ith sequence-pair in $\mathbf{D}$) that is optimal to those parameters, as follows.

Using each $\langle \mathcal{A}_i, \mathbf{S}_i, \mathbf{T}_i \rangle \in \mathbf{D}$, we want to estimate the best (integer) value of t_i for a fixed stochastic matrix $\mathbf{M}$ (i.e. $\mathbf{M}(t_i) = \mathbf{M}^{t_i}$) and associated parameters stated above, such that the total message length of stating $\mathcal{A}_i$, $\mathbf{S}_i$ and $\mathbf{T}_i$ is minimised:

$$\begin{aligned} I(\mathcal{A}_i, \langle \mathbf{S}_i, \mathbf{T}_i \rangle \mid \mathbf{M}(\text{t}), \boldsymbol{\alpha}, \boldsymbol{\Theta}) = I(\mathcal{A}_i | \boldsymbol{\alpha}, \boldsymbol{\Theta}, t_i) \; &+ \sum_{\substack{\forall \mathcal{A}_i(j) = \textbf{match} \\ \& \mathcal{A}_i(j) \implies \binom{\mathbf{S}_i(k)}{\mathbf{T}_i(l)}}} I(\mathbf{S}_i(k), \mathbf{T}_i(l) | \mathbf{M}(t_i)) \\ &+ \sum_{\substack{\forall \mathcal{A}_i(j) = \textbf{delete} \\ \& \mathcal{A}_i(j) \implies \binom{\mathbf{S}_i(k)}{-\text{ (gap)}}}} I(\mathbf{S}_i(k) | \mathbf{P}) \\ &+ \sum_{\substack{\forall \mathcal{A}_i(j) = \textbf{insert} \\ \& \mathcal{A}_i(j) \implies \binom{-\text{ (gap)}}{\mathbf{T}_i(l)}}} I(\mathbf{T}_i(l) | \mathbf{P}) \end{aligned}$$

where $\mathcal{A}_i(j)$ is the j-th state in the 3-state string $\mathcal{A}_i$, $\mathbf{S}_i(k)$ is the k-th amino acid in the sequence $\mathbf{S}_i$, and similarly for $\mathbf{T}_i(l)$.

Using the above objective function, we implemented a variant of the bisection method over the integral values of $t \in [1, 1000]$, where in each iteration, the interval is reduced by half (i.e. the search range is halved).

The variation attempts to handle some special cases to avoid the method from being trapped within in a local optima.

Finally, once t_i is inferred for $\langle \mathcal{A}_i, \mathbf{S}_i, \mathbf{T}_i \rangle$, optimal to the parameters $\mathbf{M}, \mathbf{P}, \boldsymbol{\alpha}, \boldsymbol{\Theta}$, the statement cost of integer t_i uniform in the range $[1, 1000]$ takes $\log_2(1000)$ bits to encode.

S1.3.4 Computation of $I(\boldsymbol{\alpha})$, $I(\boldsymbol{\Theta}_i|\boldsymbol{\alpha}, t_i)$, and $I(\mathcal{A}_i|\boldsymbol{\Theta}_i, \boldsymbol{\alpha}, t_i)$

The computation of these terms are feasible when the time parameters $\boldsymbol{\tau} = \{t_1, \ldots, t_{|\mathbf{D}|}\}$ for each aligned sequence-pair $\langle \mathcal{A}_i, \mathbf{S}_i, \mathbf{T}_i \rangle \in \mathbf{D}$ are known. We first discuss the relationship between Dirichlet and three-state machine models, before exploring the computation of their respective message length terms.

Relationship between Dirichlet parameters and 3-state machine transition probabilities The **Methods and Material** section in the main text introduces the alignment 3-state machine over `match` (`m`), `insert` (`i`), and `delete` (`d`) states, with 9 transition probability parameters between any two states. As shown in main text's FIG. 4, the alignment 3-state machine is symmetric between `insert` (`i`) and `delete` (`d`) states, thus yielding 3 free parameters (and remaining 6 dependent).

Notionally, the `match` state has only 1 free parameter, $\Pr(\mathtt{m}|\mathtt{m})$. The `insert` state has 2 free parameters, $\Pr(\mathtt{i}|\mathtt{i})$ and $\Pr(\mathtt{m}|\mathtt{i})$. The `delete` state is treated as symmetric to the `insert` state. Thus, remaining 6 transition probabilities can be derived from the symmetry between `insert` and `delete` states, and from the constraint that all transition probabilities out of any state in the machine add up to 1.

All 9 transition probabilities in a alignment 3-state machine can be derived from the following composition of $\mathbb{L}_1$-normalized vectors, $\vec{\Theta}^{(\mathbf{match})}$ (defining a point in 1-simplex) and $\vec{\Theta}^{(\mathbf{insert})}$ (defining a point in 2-simplex):

$$\vec{\Theta}^{(\mathbf{match})} = \left\{ \underbrace{\Pr(\mathtt{m}|\mathtt{m})}_{\text{free}}, \underbrace{1 - \Pr(\mathtt{m}|\mathtt{m})}_{\text{dependent}} \right\}$$

$$\vec{\Theta}^{(\mathbf{insert})} = \left\{ \underbrace{\Pr(\mathtt{i}|\mathtt{i})}_{\text{free}}, \underbrace{\Pr(\mathtt{m}|\mathtt{i})}_{\text{free}}, \underbrace{1 - \Pr(\mathtt{i}|\mathtt{i}) - \Pr(\mathtt{m}|\mathtt{i})}_{\text{dependent}} \right\} \equiv \vec{\Theta}^{(\mathbf{delete})} \quad \text{(by symmetry)}$$

Therefore, $\vec{\Theta}^{(\mathbf{match})}$ is a variable modelled using a 1-simplex Dirichlet distribution $\mathrm{Dir}(\vec{\alpha}^{(\mathbf{match})})$, with $\vec{\alpha}^{(\mathbf{match})}$ having $d = 2$ degrees of freedom, and $\vec{\Theta}^{(\mathbf{insert})}$ is a variable modelled using a 2-simplex Dirichlet distribution $\mathrm{Dir}(\vec{\alpha}^{(\mathbf{insert})})$, with $\vec{\alpha}^{(\mathbf{insert})}$ having $d = 3$ degrees of freedom. The MML estimates for $\vec{\Theta}^{(\mathbf{match})}$ and $\vec{\Theta}^{(\mathbf{insert})}$ are as per the general case given in Equation 8.

To infer time-dependent Dirichlet distributions, we partition the alignments in the benchmark $\mathbf{D}$ into time (t) related integer bins in the range $t \in [1, 1000]$. The subset of alignments in each time-bin is denoted as $\mathbf{A}(t)$, where $\mathbf{A}(t) \subset \mathbf{D}$.

Given each time-bin with the subset of alignments $\mathbf{A}(t)$ containing $|\mathbf{A}(t)|$ number of alignment 3-state strings, the goal is to infer the time-dependent Dirichlet parameters $\boldsymbol{\alpha}(t) = \left\{\vec{\alpha}^{(\mathbf{match})}, \vec{\alpha}^{(\mathbf{insert})}\right\}$ for that bin that model the observed set of 3-state machine parameters $\boldsymbol{\Theta}(t) = \left\{ \boldsymbol{\Theta}_i \equiv \left[\vec{\Theta}_i^{(\mathbf{match})}, \vec{\Theta}_i^{(\mathbf{insert})} \right] \right\}_{1 \le i \le |\mathbf{A}(t)|}$ inferred for each alignment $\mathcal{A}_i \in \mathbf{A}(t)$.

Message length terms We want to infer $\boldsymbol{\alpha}(t)$ and $\boldsymbol{\Theta}(t)$ using the observation of alignment states in $\mathbf{A}(t)$, by minimising the objective function:

$$I(\boldsymbol{\alpha}(t), \boldsymbol{\Theta}(t), \mathbf{A}(t)) = I(\boldsymbol{\alpha}(t)) + I(\boldsymbol{\Theta}(t)|\boldsymbol{\alpha}(t)) + I(\mathbf{A}(t)|\boldsymbol{\Theta}(t), \boldsymbol{\alpha}(t)) \tag{9}$$

where the terms on the right-hand side can be further decomposed as:

$$I(\boldsymbol{\alpha}(t)) = I(\vec{\alpha}^{(\mathbf{match})}) + I(\vec{\alpha}^{(\mathbf{insert})}) \tag{10}$$

$$I(\boldsymbol{\Theta}|\boldsymbol{\alpha}(t)) = \sum_{i=1}^{|\mathbf{A}(t)|} \left(I(\vec{\Theta}_i^{(\mathbf{match})}|\vec{\alpha}^{(\mathbf{match})}, t_i = t) \; + \; I(\vec{\Theta}_i^{(\mathbf{insert})}|\vec{\alpha}^{(\mathbf{insert})}, t_i = t) \right) \tag{11}$$

$$I(\mathbf{A}(t)|\boldsymbol{\Theta}, \boldsymbol{\alpha}(t)) = \sum_{i=1}^{|\mathbf{A}(t)|} I(\mathcal{A}_i|\vec{\Theta}_i, \boldsymbol{\alpha}, t_i = t) \tag{12}$$

Further, each state-related Dirichlet parameter in the right-hand side of Eq. 10 of the form $\vec{\alpha}^{(\langle \mathbf{state} \rangle)}$ where $\langle \mathbf{state} \rangle \in \{\mathbf{match}, \mathbf{insert}\}$, expands to the form (using method in [33]):

$$I(\vec{\alpha}^{(\langle \mathbf{state} \rangle)}) = \frac{1}{2} \log \left(1 + \frac{det[Fisher(\vec{\alpha}^{(\langle \mathbf{state} \rangle)})] \times (c_d)^d}{h(\vec{\alpha}^{(\langle \mathbf{state} \rangle)})^2} \right) + \frac{d}{2} \tag{13}$$

In the above expansion, $h(\vec{\alpha}^{(\langle\textbf{state}\rangle)})$ is the prior on the state-related Dirichlet parameters $\vec{\alpha}^{(\langle\textbf{state}\rangle)}$. c_d is the lattice constant [8] associated with d degrees of freedom – recall, $d = 2$ for Dirichlet associated with the `match` state, and $d = 3$ for `insert` state; $c_2 = \frac{5}{36\sqrt{3}}$ and $c_3 = \frac{19}{192\times 2^{\frac{1}{3}}}$. Finally, $det[Fisher(\vec{\alpha}^{(\langle\textbf{state}\rangle)})]$ is the determinant of the expected Fisher information of $\vec{\alpha}^{(\langle\textbf{state}\rangle)}$ given in Eq. 4. For details of the prior used, see [29, supplementary methods].

We note that the overall statement length of encoding all "time"-dependent Dirichlet parameters for each time-bin ($\forall t \in [1, 1000]$) is the sum of their individual bin-wise statement costs: $I(\boldsymbol{\alpha}) = \sum_{t=1}^{1000} I(\boldsymbol{\alpha}(t))$.

Similarly, each state-related transition probability parameters in the right-hand side of Eq. 11 of the form $I(\vec{\Theta}^{(<\textbf{state}>)}|\vec{\alpha}^{(\langle\textbf{state}\rangle)})$ (again $\langle\textbf{state}\rangle \in \{\textbf{match}, \textbf{insert}\}$) expands to (using the methods in [33]):

$$I(\vec{\Theta}_i^{(\langle\textbf{state}\rangle)}|\vec{\alpha}^{(\langle\textbf{state}\rangle)}) = \sum_{i=1}^{|\mathbf{A}(t)|} \left(\frac{1}{2} \log \left(1 + \frac{det[Fisher(\vec{\Theta}_i^{(\langle\textbf{state}\rangle)})]\,(c_{d-1})^{d-1}}{(\mathrm{Dir}(\vec{\Theta}_i^{(\langle\textbf{state}\rangle)}; \vec{\alpha}^{(\langle\textbf{state}\rangle)}))^2} \right) + \frac{d-1}{2} \right) \tag{14}$$

The determinant of the expected Fisher information of the inferred MML probability estimates $\vec{\Theta}^{(\langle\textbf{state}\rangle)} = (\theta_1, \theta_2, \ldots, \theta_d)$ (see Eq. 8), with $d-1$ free parameters (notionally, with dependent parameters $\theta_d = 1 - \sum_{i=1}^{d-1} \theta_i$) on each alignment $\mathcal{A}_i \in \mathbf{A}$ is given by:

$$det[Fisher(\vec{\Theta}_i^{(\langle\textbf{state}\rangle)})] = \frac{(counts(\langle\textbf{state}\rangle \longrightarrow \{\texttt{match}, \texttt{insert}, \texttt{delete}\}))^{d-1}}{\theta_i \theta_2 \cdots \theta_d}$$

where $counts(\langle\textbf{state}\rangle \longrightarrow \{\texttt{match}, \texttt{insert}, \texttt{delete}\})$ is the number of observed transitions from the specified state to any of the {`match`, `insert`, `delete`} states in alignment $\mathcal{A}_i$. Specifically, when $\langle\textbf{state}\rangle$ = `match` we count the total number of `m`→`m`, `m`→`i` and `m`→`d` transitions in $\mathcal{A}_i$, while the denominator refers to the product of $d = 2$ MML estimates for Pr(`m`|`m`) and (1-Pr(`m`|`m`)). Similarly, when $\langle\textbf{state}\rangle$ = `insert` (which is symmetric with `delete` state), we count the total number of `i`→`i` plus `d`→`d`, `i`→`m` plus `d`→`m`, `i`→`d` plus `d`→`i` transitions observed in $\mathcal{A}_i$, while the denominator refers to the product of $d = 3$ MML estimates for Pr(`i`|`i`), Pr(`m`|`i`) and (1-Pr(`i`|`i`)- Pr(`m`|`i`) = Pr(`d`|`i`)).

Finally, the statement of each 3-state string $\mathcal{A}_i$ in the time-bin $\mathbf{A}(t)$, given on the right-hand side of Eq. 12, can be expanded to:

$$I(\mathcal{A}_i|\vec{\Theta}_i, \boldsymbol{\alpha}, t_i = t) = \sum_{j=1}^{|\mathcal{A}_i|} -\log(\Pr(\mathcal{A}_i(j)|\mathcal{A}_i(j-1))$$

where $\mathcal{A}_i(j)$ is the j-th character in the 3-state string $\mathcal{A}_i$ whose length is $|\mathcal{A}_i|$, and Pr(.|.) is one of the 9 transition probabilities of the 3-state machine associated with the alignment $\mathcal{A}_i$, derived from $\vec{\Theta}_i$.

S1.4 Monte Carlo search to infer all parameters over a given benchmark D

Using the various encoding length terms in Section 1.3, we rely on a standard Expectation-Maximisation (EM) [11] like strategy to simultaneously infer optimal parameters across all statistical models required to estimate the Shannon information content of any collection $\mathbf{D}$ (under those models).

Broadly, the approach starts from any specified initial values for $\mathbf{M}(t)$ and $\boldsymbol{\alpha}(t)$. A good starting point would be from any of the existing substitution matrices (after converting to their stochastic base ($t = 1$) matrix form). Similarly $\boldsymbol{\alpha}(t)$ inferred from any of the earlier matrices is a good starting point. The files containing our starting points are available here: `https://lcb.infotech.monash.edu.au/mmlsum`.

Note, as stated above in Section S1.3.2, the optimal MML estimates of $\mathbf{P}$ is solely dependent on the benchmark $\mathbf{D}$ and not on any other parameters.

The EM-like search involves holding $\boldsymbol{\alpha}(t)$ and $\vec{\Theta}$ fixed while performing a Monte Carlo search (with standard simulated annealing [17]) to optimise for $\mathbf{M}$ and $\boldsymbol{\tau}$. (These details are presented in Section S1.4.2) After this, $\mathbf{M}$ and $\boldsymbol{\tau}$ are held fixed to estimate $\boldsymbol{\alpha}(t)$ and $\vec{\Theta}$. (These details are presented in S1.4.1). This process is repeated until the objective function converges.

S1.4.1 Search for time-dependent Dirichlet parameters with fixed values of M and $\boldsymbol{\tau}$

Holding the stochastic matrix $\mathbf{M}$ and time parameters $\boldsymbol{\tau}$ fixed, this allows us to partition all alignments in the benchmark $\mathbf{D}$ into respective time-bins, resulting in subsets of alignments $\mathbf{A}(t)$ for each $t \in [1, 1000]$.

There is no closed-form to estimate the parameters of a Dirichlet distribution. For each $t \in [1, 1000]$, starting from the current state of $\alpha(t)$, we iteratively perform random perturbations on $\alpha(t)$ in its near-neighborhood by either perturbing the mean vector or the concentration parameter (see pseudocode in Figure SF1).

Specifically, in each iteration, there is 50-50 choice between `Perturb_Mean` and `Perturb_Concentration` functions (refer decomposition of Dirichlet into mean and concentration given in Section S1.2). The former perturbs the mean vector $\hat{\mu}$ of $\vec{\alpha}^{(\langle\textbf{state}\rangle)}$ by sampling a new probability vector from a Dirichlet distribution of mean $\vec{\alpha}^{(\langle\textbf{state}\rangle)}$ and a specified concentration $\bar{\kappa}$. The latter randomly either increases or decreases the concentration parameter by $\pm\delta$ from its current value.

```
Function Perturb_Dirichlet_Param(α⃗^(⟨state⟩)):
    {κ,μ̂} ← decompose(α⃗^(⟨state⟩));
    u1 ← random_uniform(0, 1);
    if u1 ≤ 0.5 then
        μ̂ ← Perturb_Mean(μ̂,⟨state⟩);
    end
    else
        κ ← Perturb_Concentration(κ);
    end
return κ.μ̂

Function Perturb_Mean(μ̂,⟨state⟩):
    if ⟨state⟩ = match then
        κ̄ ← 10000;
    end
    else
        κ̄ ← 1000;
    end
return sample_dirichlet(κ̄.μ̂)

Function Perturb_Concentration(κ):
    δ ← random_uniform(0.1, 10);
    u2 ← random_uniform(0, 1);
    if u2 ≤ 0.5 then
        κ ← κ + δ;
    end
    else
        κ ← κ - δ;
    end
return κ

Function sample_dirichlet(α⃗):
    Init y⃗ ← 0⃗, sum ← 0 ;
    for (i = 1 → |α⃗|) do
        y_i ← gamma_random(α⃗_i, 1);
        sum ← sum + y_i;
    end
    for (i = 1 → |α⃗|) do
        y_i ← y_i/sum ;
    end
return y⃗
```

Figure SF1: Pseudocode for Dirichlet parameter perturbation

After each perturbation, the message length defined in Equation 9 is evaluated. If the message length decreases, it is accepted with a probability of 1, else the perturbation is accepted/rejected with a probability equivalent to $2^{-\Delta I}$, where ΔI is the observed difference in the message lengths (in bits).

At the end of this process, $\boldsymbol{\alpha}(t), \forall t \in [1, 1000]$ are optimised for the fixed values of $\mathbf{M}$ and $\boldsymbol{\tau}$. $\boldsymbol{\Theta}$ are estimated from these new values of $\boldsymbol{\alpha}(t)$ as described in Section S1.3.4.

S1.4.2 Search for the best stochastic matrix with fixed values of $\boldsymbol{\alpha}$ and $\boldsymbol{\Theta}$

While holding the Dirichlet and 3-state machine parameters ($\boldsymbol{\alpha}$ and $\boldsymbol{\Theta}$) fixed, the current state of the stochastic (base $t = 1$) matrix is optimised over all set of sequence-pairs and their alignments in the benchmark $\mathbf{D}$, using a simulated annealing approach.

Starting from an initial temperature parameter of $temp = 10000$, we gradually cool down the system using a cooling schedule where the temperature is decreased by a factor of 0.88, after 500 perturbations (described below) of the stochastic matrix at each temperature mark. The process continues until 0.0001 temperature.

In each perturbation of $\mathbf{M}$, a column of the current matrix is randomly selected according to its stationary distribution. The perturbation of the selected column vector is done by sampling from a Dirichlet distribution with the column vector used as the mean $\hat{\mu}$ and with a specified high concentration parameter κ. The pseudocode of the function `sample_dirichlet`($\vec{\alpha} = \hat{\mu}\kappa$) has been previously shown in Figure SF1. Initially κ is set to $1,000,000$. As the simulated annealing cools (by a factor of 0.88), the distribution is made even more concentrated by increasing the κ by a factor of $\frac{1}{0.88}$, thus making the neighbourhood of sampling tighter and tighter as the simulated annealing cools and the matrix $\mathbf{M}$ converges.

For each perturbation of $\mathbf{M} \to \tilde{\mathbf{M}}$, the total lossless encoding length as per Equation 2 (see main text **Methods and Material** section) is recomputed using $\tilde{\mathbf{M}}$. The matrix is accepted or rejected based on the Metropolis criterion: if the encoding length decreases, then the change is accepted with a probability of 1; Otherwise, it is accepted with a probability of $2^{-\frac{\Delta I}{temp}}$, where ΔI is the difference in the encoding lengths using $\tilde{\mathbf{M}}$ versus $\mathbf{M}$ in bits.

S1.5 Additional information

S1.5.1 Computing the expected change of a stochastic matrix

The expected change of a stochastic matrix modelling amino acid substitutions gives the probability of observing a change in any amino acid state on average. In any column of the matrix $\mathbf{M}(t)$ indexed by j, the diagonal element $\mathbf{M}_{jj}(t)$ represents the probability of observing a *conservation* (i.e. no change) in the corresponding amino acid state, under the substitution model that $\mathbf{M}$ specifies. The weighted average of these terms (i.e.

weighted by the stationary probability π_j of each amino acid) over all columns, $\sum_{j=1}^{20} \pi_j \mathbf{M}_{jj}(t)$, gives the expected probability of amino acids being conserved under the stochastic model $\mathbf{M}(t)$. Thus:

$$\text{Expected } change = 1.0 - \sum_{j=1}^{20} \pi_j \mathbf{M}_{jj}(t)$$

S1.5.2 Converting a log-odds scoring matrix into a stochastic matrix

The approach of establishing a common ground for a fair and consistent comparison between various substitution matrices was made on the basis of converting all non-Markov models into Markov models. Following describes the criteria used for this transformation.

1. Convert a published, log odds scoring matrix S into its conditional probability matrix form using the following relationship:

$$S_{ij} = c.\log_2 \left[\frac{\Pr(a_i, a_j)}{\Pr(a_i)\Pr(a_j)} \right] = c.\log_2 \left[\frac{\Pr(a_i|a_j)}{\Pr(a_i)} \right] = c.\log_2 \left[\frac{\Pr(a_j|a_i)}{\Pr(a_j)} \right] \tag{15}$$

   where a_i and a_j are the amino acids indexed by i and j respectively; c is the scaling factor to set the unit of information as $\frac{1}{c}$ bits (e.g. $c = 2$ implies half bit unit; $c = 3$ implies third bit unit). In case the original amino acid null frequencies are absent, we used the 20-nomial model ($\mathbf{P}$) which is optimal under the `SCOP2` benchmark in order to include it in the fairest way possible.

2. If the available/derived substitution probability matrix is not reflecting a 0.01 expected change, assume it as representing some $\mathbf{M}(\mathrm{t})$ of a Markov model and derive its approximate base matrix $\tilde{\mathbf{M}}(1)$ by finding the kth matrix root which is the closest to 0.01 expected change, which is then normalized.

S2 Supplementary Results

S2.1 Distribution of sequence-identity in the benchmarks

The figure below gives the sequence-identity distribution of alignments in each of the six benchmarks we considered. This accompanies the discussion of benchmarks in the **Results and Discussion** section of the main text.

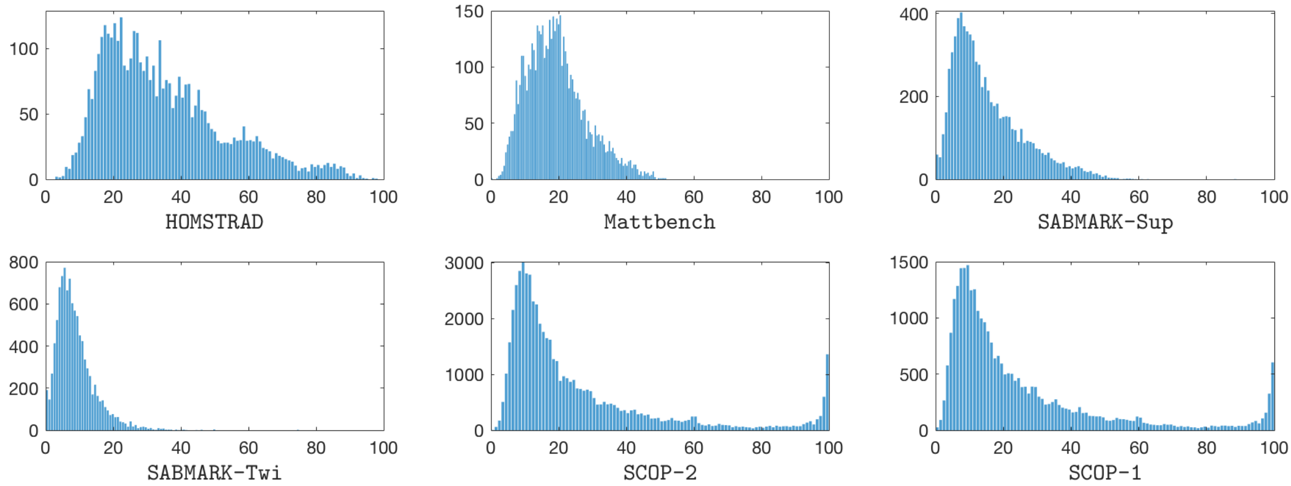

Figure SF2: Sequence identity percentage distributions (histograms) across the benchmarks

S2.2 Extended Analysis accompanying results in the main text

Shannon information content across matrices and benchmarks by varying P. Table 2 of the main text **Results** section examined the measure of Shannon information content by varying the substitution matrix model, while all other parameters (including the 20-nomial probabilities $\mathbf{P}$) were optimised for the respective matrices and benchmarks.

As an extended analysis, we explore two other alternatives for $\mathbf{P}$ to study their effect on the rank order, although they turned out to be suboptimal compared to the optimal ones reported in the main text, Table 2. These alternatives are:

1. inferring a 20-nomial from an independent-source – here, we use the entire set of non-redundant protein sequences in the UniProt database [7];

2. using the stationary distribution of the stochastic matrix **M** (i.e. derived from the eigenvector of **M** associated with the eigenvalue of $\lambda = 1$).

Table ST1 shows the Shannon information content across the matrices and benchmarks, for the first choice enumerated above. We observe that, although the encoding lengths are longer (worser), the effect on the rank order is minimal. The order (based on `ranksums`) observed in Table 2 of the main text are generally preserved, barring `LG` and `MIQS` where their ranks over `SCOP2` have swapped over. In comparison, Table ST2 shows the same encoding lengths using the second choice enumerated above, and there are some changes to the rank order, although $\mathrm{MML}_{\texttt{SCOP2}}$ matrix still performs the best across all benchmarks with the lowest `ranksum=16`.

Table ST1: Shannon information content to losslessly encode each benchmark with varying substitution matrices along with their ranks (reported inside brackets), when using the UniProt based MML estimates as the amino acid 20-nomial distribution **P**.

Matrix	HOMSTRAD	Mattbench	SABMARK-Sup	SABMARK-Twi	SCOP-1	SCOP-2
Shannon information using existing substitution models						
PAM (1978)	11547347.32 (15)	9155559.27 (15)	23628852.13 (15)	11336736.12 (15)	85026098.24 (15)	82893663.55 (15)
JTT (1992)	11496994.08 (14)	9084491.06 (13)	23505598.27 (13)	11278424.47 (13)	84454677.89 (13)	82354250.08 (13)
BLOSUM (1992)	11453343.72 (10)	9049472.26 (08)	23428674.77 (07)	11254553.75 (06)	84275402.19 (11)	82130897.40 (10)
JO (1993)	11492309.39 (13)	9130688.40 (14)	23556128.16 (14)	11316566.45 (14)	84668169.16 (14)	82541280.05 (14)
WAG (2001)	11434976.91 (05)	9065145.32 (12)	23454783.64 (11)	11269752.08 (12)	84242325.21 (09)	82131872.38 (11)
VTML (2002)	11439289.05 (07)	9048325.84 (07)	23432271.63 (08)	11257134.26 (08)	84176599.86 (07)	82061020.74 (07)
LG (2008)	11480054.48 (12)	9061463.01 (11)	23466479.90 (12)	11261899.34 (09)	84356348.28 (12)	82226007.09 (12)
MIQS (2013)	11438006.26 (06)	9052903.26 (10)	23440009.45 (10)	11262833.45 (10)	84177434.21 (08)	82063425.69 (08)
PFASUM (2017)	11428679.05 (02)	9052221.77 (09)	23433841.03 (09)	11263082.39 (11)	84141210.67 (04)	82038431.69 (06)
Shannon information using MML inferred substitution models						
$\mathrm{MML}_{\texttt{HOMSTRAD}}$	11421395.62 (01)	9047739.99 (05)	23419917.83 (06)	11256695.07 (07)	84126993.63 (03)	82009294.02 (03)
$\mathrm{MML}_{\texttt{MATTBENCH}}$	11442135.03 (09)	9038304.82 (01)	23409982.48 (03)	11243729.20 (03)	84151618.78 (05)	82022514.41 (04)
$\mathrm{MML}_{\texttt{SABMARK-Sup}}$	11439926.80 (08)	9043738.29 (04)	23400792.07 (01)	11238762.79 (02)	84168584.28 (06)	82024870.38 (05)
$\mathrm{MML}_{\texttt{SABMARK-Twi}}$	11458572.58 (11)	9048142.90 (06)	23410821.13 (05)	11237871.03 (01)	84256392.91 (10)	82098025.94 (09)
$\mathrm{MML}_{\texttt{SCOP1}}$	11429086.52 (03)	9042105.23 (03)	23410002.48 (04)	11247805.78 (05)	84097487.36 (01)	81984099.12 (02)
$\mathrm{MML}_{\texttt{SCOP2}}$	11429516.19 (04)	9041089.95 (02)	23404593.45 (02)	11244715.72 (04)	84100227.75 (02)	81976372.74 (01)

Table ST2: Shannon information content to losslessly encode each benchmark with varying substitution matrices along with their ranks (reported inside brackets), when using the matrix stationary distribution as the amino acid 20-nomial distribution **P**.

Matrix	HOMSTRAD	Mattbench	SABMARK-Sup	SABMARK-Twi	SCOP-1	SCOP-2
Shannon information using existing substitution models						
PAM (1978)	11547416.21 (15)	9164067.18 (15)	23657379.78 (15)	11355479.09 (15)	85072048.36 (15)	82949452.90 (15)
JTT (1992)	11496340.10 (14)	9084284.48 (13)	23499066.75 (13)	11273707.07 (11)	84446694.25 (13)	82346379.77 (13)
BLOSUM (1992)	11459479.32 (11)	9060438.92 (10)	23466561.57 (11)	11275166.62 (13)	84354008.80 (11)	82232019.26 (11)
JO (1993)	11490030.65 (13)	9139874.62 (14)	23571493.08 (14)	11328875.88 (14)	84680102.91 (14)	82560968.37 (14)
WAG (2001)	11432008.93 (07)	9065038.48 (11)	23442803.68 (10)	11262564.43 (10)	84218617.83 (10)	82107643.24 (10)
VTML (2002)	11439908.46 (10)	9049441.74 (07)	23432043.81 (09)	11255766.35 (09)	84180147.95 (09)	82070311.94 (09)
LG (2008)	11485493.34 (12)	9065759.17 (12)	23491875.49 (12)	11274788.00 (12)	84399024.43 (12)	82286691.96 (12)
MIQS (2013)	11435028.94 (08)	9051333.90 (09)	23421817.75 (08)	11252372.16 (08)	84147813.60 (08)	82031386.28 (08)
PFASUM (2017)	11423630.07 (02)	9048568.06 (06)	23406776.08 (05)	11248125.05 (06)	84095385.87 (04)	81984520.86 (04)
Shannon information using MML inferred substitution models						
$\mathrm{MML}_{\texttt{HOMSTRAD}}$	11418789.53 (01)	9050681.63 (08)	23408123.19 (06)	11250120.55 (07)	84113701.63 (06)	82002419.36 (06)
$\mathrm{MML}_{\texttt{MATTBENCH}}$	11436960.04 (09)	9040249.80 (01)	23416280.31 (07)	11248122.03 (05)	84130366.58 (07)	82027458.07 (07)
$\mathrm{MML}_{\texttt{SABMARK-Sup}}$	11423841.26 (03)	9044793.01 (05)	23401281.01 (01)	11244464.40 (02)	84092356.97 (03)	81982211.04 (03)
$\mathrm{MML}_{\texttt{SABMARK-Twi}}$	11427701.22 (06)	9043437.94 (02)	23404974.85 (04)	11243567.19 (01)	84099460.19 (05)	81990981.18 (05)
$\mathrm{MML}_{\texttt{SCOP1}}$	11424651.38 (05)	9043895.49 (04)	23403924.44 (03)	11244987.34 (03)	84075928.68 (01)	81970279.92 (02)
$\mathrm{MML}_{\texttt{SCOP2}}$	11423906.32 (04)	9043878.06 (03)	23403244.25 (02)	11245052.04 (04)	84077082.89 (02)	81968197.28 (01)

S3 Additional information

S3.1 Analysis of encoding lengths using BLOSUM and PFASUM series of matrices

Here we analyse how all matrices in the BLOSUM series (most popularly used substitution matrix series) and PFASUM series (the best-performing, BLOSUM-like series among the existing set of substitution matrices compared in this work) perform in compressing the `SCOP2` benchmark.

In Table 2 of the main text, the rows reporting the encoding lengths for BLOSUM and PFASUM were by using the converted forms of BLOSUM62 and PFASUM60 matrices respectively. (These were chosen as they are the reported general-purpose log-odds scoring matrices in their respective series.) Table ST3 extends this

analysis to other possible matrices in their series, all compressing the same `SCOP2` benchmark. The accompanying Figure SF3 illustrates the total encoding message lengths (i.e. Shannon information content) reported in Table ST3 as a function of the expected amino acid change implicit in their original scoring matrices.

Consistent with the picture that emerged when comparing existing matrices, we observe (see Figure SF3) that the PFASUM series performs better than BLOSUM series. Across the matrices in PFASUM, we observe a flat trend of encoding lengths suggesting their internal consistency, and that any of them are equally good to derive a stochastic matrix from. On the other hand, BLOSUM series, with some variations, has more or less a flat trend when using matrices converted into a stochastic form within the range [45,100], whereas matrices in the range [30,40] give substantially worse (larger) message lengths in comparison. This suggests that BLOSUM[30-40] series of matrices do not generalise well to explain the range of relationships in `SCOP2` benchmark.

Table ST3: Lossless encoding lengths (in bits) resulted from using various substitution matrices from the BLOSUM and PFASUM series, to compress the `SCOP2` benchmark.

BLOSUM series	Original expected change	Total message length
BLOSUM30	0.8482	82439452.76
BLOSUM35	0.8161	82227426.03
BLOSUM40	0.7903	82060377.07
BLOSUM45	0.7587	81999170.77
BLOSUM50	0.7286	81966740.66
BLOSUM55	0.7041	81964908.57
BLOSUM60	0.6790	81981508.57
BLOSUM62	0.6681	81995179.31
BLOSUM65	0.6530	81994670.34
BLOSUM70	0.6309	82004034.57
BLOSUM75	0.6128	82014056.31
BLOSUM80	0.5928	82010530.24
BLOSUM85	0.5684	82012220.79
BLOSUM90	0.5413	81997158.82
BLOSUM95	0.5129	81999074.38
BLOSUM100	0.4729	82004612.48

PFASUM series	Original expected change	Total message length
PFASUM30	0.8219	81910293.61
PFASUM35	0.8059	81904481.06
PFASUM40	0.7896	81901816.25
PFASUM45	0.7739	81899988.62
PFASUM50	0.7584	81900015.41
PFASUM55	0.7427	81901163.14
PFASUM60	0.7268	81902713.60
PFASUM62	0.7204	81903374.10
PFASUM65	0.7108	81904599.34
PFASUM70	0.6946	81906335.59
PFASUM75	0.6774	81907860.48
PFASUM80	0.6562	81909272.54
PFASUM85	0.6540	81909221.82
PFASUM90	0.6538	81909091.84
PFASUM95	0.6537	81909179.07
PFASUM100	0.6596	81920990.78

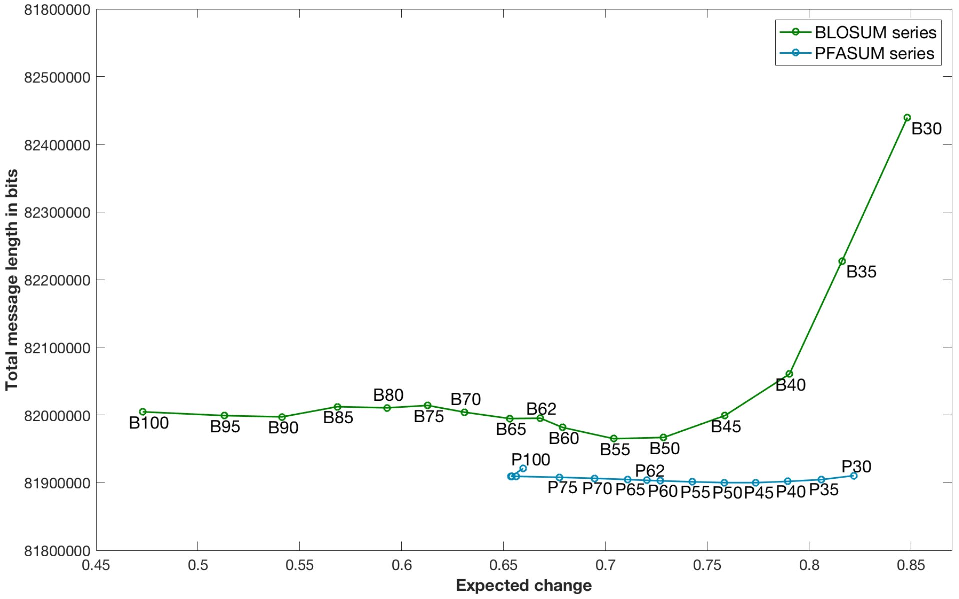

Figure SF3: Lossless encoding lengths resulted from using matrices in the BLOSUM and PFASUM series to compress `SCOP2` benchmark, plotted against their original expected change of amino acids.

S3.2 Kullback-Leibler (KL) divergence between matrices

We now undertake an analysis to quantitatively highlight the similarities and differences between ten stochastic matrices (the nine existing matrices and MMLSUM (i.e. $\mathrm{MML}_{\texttt{SCOP2}}$)) independent of any benchmarks. We emphasise that this analysis does *not* provide any statement on the relative performance of various matrices (as was carried out in the main text and previous section), but will assist in gauging their relative concordance.

This analysis uses the notion of Kullback-Leibler (KL) divergence [19] computed between any two substitution matrices. KL-divergence estimates the measure of *relative* Shannon entropy between two probability

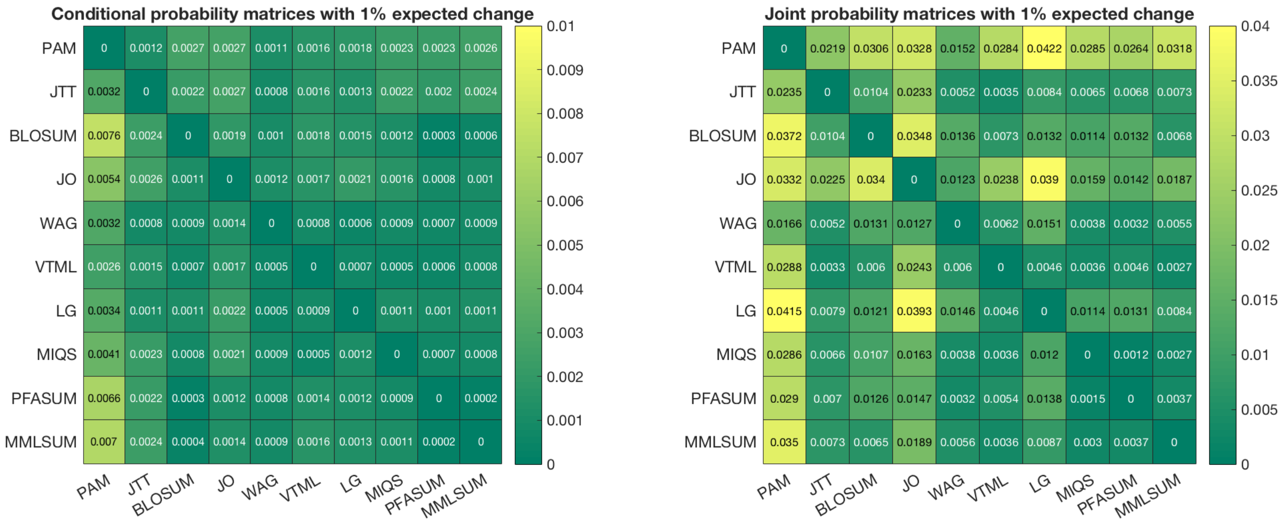

Figure SF4: KL divergence based distance matrices (in the form of heat maps) between 10 matrices (9 existing and 1 inferred using MML). Left column is the computation of KL-divergence over conditional probabilities of amino acid interchanges coming under the base matrix of 1% expected change. Right column is the computation of KL-divergence over the corresponding joint probabilities of interchanges.

distributions. For any two matrices X and Y (considered in their stochastic form), their KL-divergence is measured in two different ways as follows.

$$\text{KL divergence (over joint probabilities)} = \sum_{i=1}^{20}\sum_{j=1}^{20} X_{i,j} \log\left(\frac{X_{i,j}}{Y_{i,j}}\right)$$

$$\text{KL divergence (over conditional probabilities)} = \sum_{i=1}^{20}\sum_{j=1}^{20} X_{i,j} \log\left(\frac{X_{i|j}}{Y_{i|j}}\right)$$

where $X_{i,j}$ denotes the joint probability implied by the matrix X for the pair of amino acids indexed by i and j (similarly, $Y_{i,j}$), $X_{i|j}$ denotes the conditional probability implied by the matrix X of an amino acid indexed by i given (i.e. interchanging/substituting) an amino acid indexed by j (similarly, $Y_{i|j}$). Note, by default, the stochastic matrix is in its conditional form containing conditional probability terms between amino acids. The joint probability terms can be computed as a product of its conditional probability times its stationary probability.

Figure SF4 shows the KL-divergence – for conditional probabilities in left column; for joint probabilities in right column – between all matrices when they show an expected amino acid change of 1%. (Note, KL-divergence is not a metric, so the resultant table is not symmetric.)

S3.3 Stochastic matrix convergence to steady-state probabilities

The optimal evolutionary distance t between two amino acid sequences may vary under different substitution models. Figure SF5 illustrates the histograms of t resultant under different stochastic matrices when encoding the `SCOP2` benchmark.

Fore each matrix, the convergence of amino acids to their respective stationary probabilities is shown in Figure SF6. Here, the KL divergence was measured between each amino acid column of a stochastic (conditional probability) matrix and the matrix's stationary distribution. Once an amino acid x column reaches the steady state, the matrix has a probability of interchange of x with any amino acid, that is indistinguishable from its stationary probability.

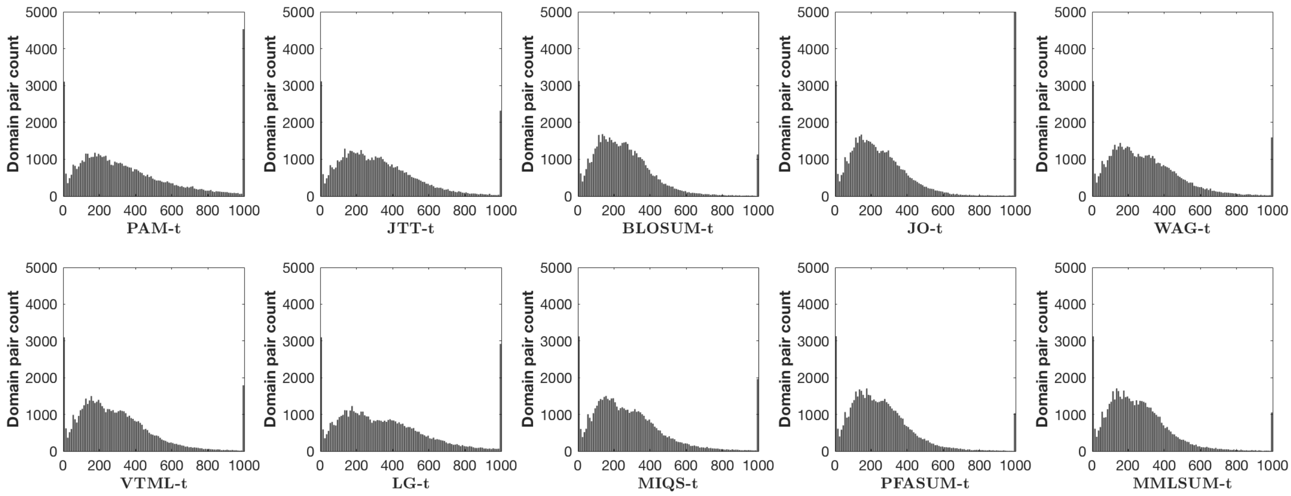

Figure SF5: Histograms of the sequence-divergence parameter t under each substitution model, inferred for the $59,092$ protein domain pairs in the `SCOP2` benchmark

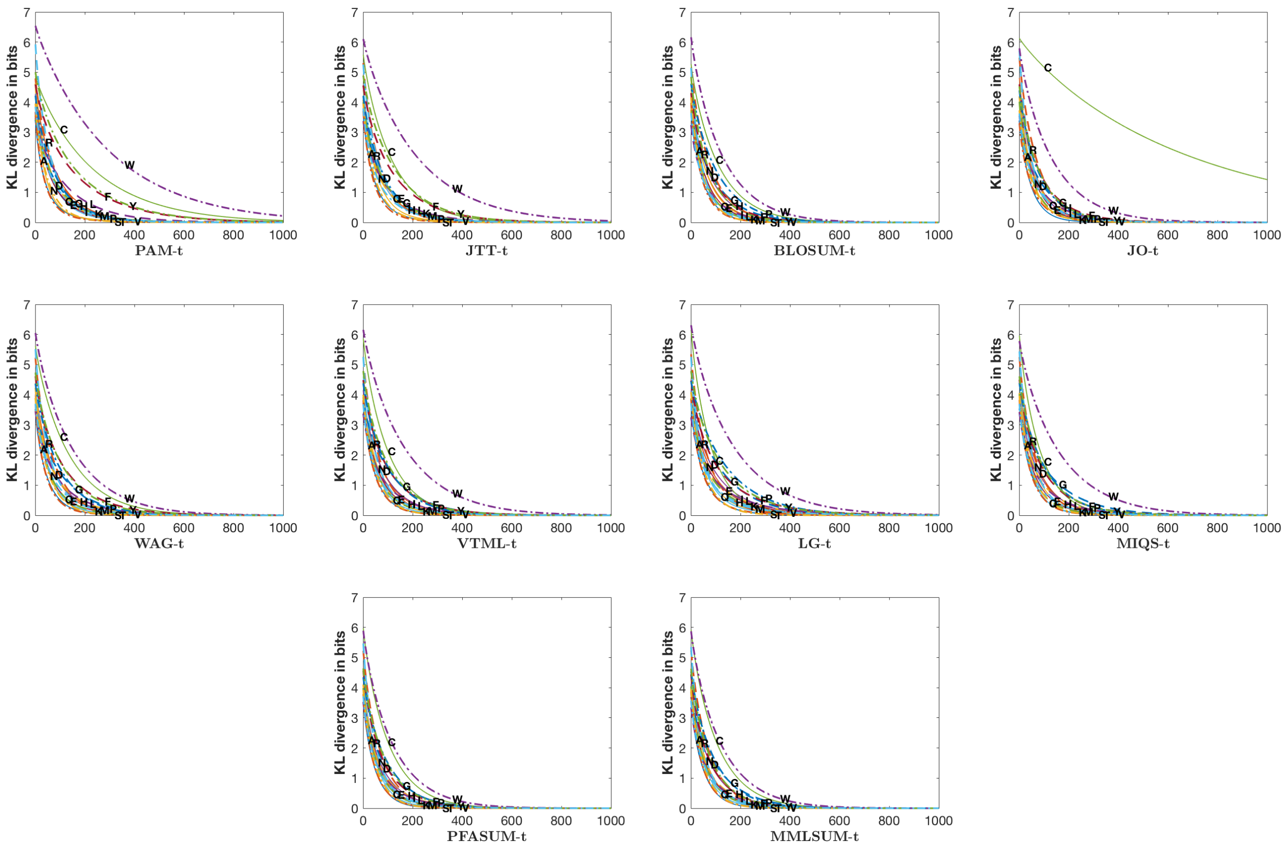

Figure SF6: The KL-divergence measuring the convergence of each column vector of various matrices to their respective stationary probabilities.

S3.4 Empirical justification for approximating divergence time with discrete-time Markov models

	HOMSTRAD	MATTBENCH	SABMARK-Sup	SABMARK-Twi	SCOP1	SCOP2
LG	t=[128,129] exp-change= [0.648,0.650]	t=[242,243] exp-change= [0.806,0.807]	t=[308,309] exp-change= [0.848,0.848]	t=[602,603] exp-change= [0.916,0.916]	t=[183,184] exp-change= [0.744,0.745]	t=[189,190] exp-change= [0.752,0.753]
WAG	t=[123,124] exp-change= [0.648,0.651]	t=[224,225] exp-change= [0.806,0.807]	t=[280,281] exp-change= [0.848,0.849]	t=[513,514] exp-change= [0.916,0.916]	t=[173,174] exp-change= [0.745,0.746]	t=[178,179] exp-change= [0.752,0.753]
MMLSUM	t=[118,119] exp-change= [0.649,0.651]	t=[208,209] exp-change= [0.806,0.807]	t=[256,257] exp-change= [0.848,0.849]	t=[452,453] exp-change= [0.916,0.916]	t=[163,164] exp-change= [0.745,0.746]	t=[167,168] exp-change= [0.751,0.753]

Figure SF7: This shows how insignificantly the 'expected-amino-acid-change' (exp-change) of a few matrices (LG, WAG and our MMLSUM) varies in the interval of $[t, t+1]$ on average, for alignments across the six benchmarks:

S3.5 Case study

In this case study we consider 9 protein chains belonging to the Globin fold:

1HHO (chain A) Oxyhemoglobin from *Homo sapiens* (Human)

2DHB (chain A) Deoxyhemoglobin from *Equus caballus* (Horse)

1MBD (chain A) Deoxymyoglobin from *Physeter catodon* (Sperm Whale)

2LH7 (chain A) Leghemoglobin from *Lupinus luteus* (Plant)

1ECO (chain A) Erythrocruorin from *Chironomus thummi thummi* (Midge)

4VHB (chain A) Hemoglobin from *vitreoscilla stercoraria* (Bacteria)

1IDR (chain A) Truncated-hemoglobin from *Mycobacterium tuberculosis* (Actinobacteria)

1DLW (chain A) Hemoglobin from *Paramecium caudatum* (Paramecium)

1DLY (chain A) Hemoglobin from *Chlamydomonas moewusii* (Green Algae)

The table below presents the inferred optimal evolutionary divergence time and compression statistics under our MMLSUM substitution model when aligning Human Hemoglobin (1HHO) against the 8 other homologous globins.

Comparison	Inferred time t_optimal	Expected a.a. change (%)	Compression -- optimal model (bits)	Compression -- marginal model (bits)
1HHO vs 2DHB	14	13	432.63	432.71
1HHO vs 1MBD	157	73	38.60	42.79
1HHO vs 2LH7	191	79	2.77	15.25
1HHO vs 4VHB	213	81	-0.10	12.08
1HHO vs 1ECO	217	82	-4.73	7.70
1HHO vs 1IDR	219	82	-13.99	1.50
1HHO vs 1DLW	223	82	-9.06	5.57
1HHO vs 1DLY	286	87	-18.75	3.98

Notice the automatically inferred time parameter (second column) correlates closely with the evolutionary distance of each species with respect to Humans (first column). Third column gives the expected amino acid change of that alignment relationship. In attempting to find the best single alignment relationship between pairs of sequences, the compression statistic (with respect to null model) is able to recognize an acceptable relationship only between human hemoglobin with horse, sperm whale and plant globins (Green entries in the fourth column), but not for distantly related globins (red entries in the fourth column). However, under the

marginal model of sequence relationship [29], all globin sequences beat the null model (green entries in the last column) and, thus, allows the inference of homology between human hemoglobin and the rest of the globin molecules.

S4 Other Supplementary Information

Following describes the six structural alignment benchmarks used in this study.

1. HOMSTRAD [22] (`https://mizuguchilab.org/homstrad`) is a database of structural alignments for homologous protein families. It contains multiple alignments of proteins covering 1032 families with known structures. Their alignments are semi-manually curated using the structural alignment programs: MNYFIT, STAMP and COMPARER [26, 27, 30].
2. Mattbench [9] (`https://bcb.cs.tufts.edu/mattbench/Mattbench.html`) is a database curated using the structural alignment program MATT [21]. This work combines its two sets of alignments classified as *superfamily* and *twilight zone* into a single benchmark. The superfamily set covers 225 groups of homologous protein domains, where all pairs of domains in any group have a sequence identity $< 50\%$. The twilight zone set is a much smaller and distinct set containing alignments covering 34 distantly related groups, where the sequence identity threshold is $< 20\%$ [9].
3. SABMARK [32] (`http://bioinformatics.vub.ac.be/databases/databases.html`) is a more extensive set of alignments covering *superfamily* and *twilight zone* protein domain sets, whose alignments are curated using SOFI and CE [5, 28]. Superfamily set (`SABMARK-sup`) contains 425 groups of multiple alignments, while the twilight zone set (`SABMARK-twi`) contains 209 groups.
4. SCOP [2] (`https://scop.berkeley.edu`) database (v2.07) was used to derive a set of 59,092 unique protein domain pairs, randomly sampled from the superfamily (36,372) and family (22,720) levels of its hierarchy. These 59,092 pairs were aligned separately using DALI [13] and MMLigner [6] to provide `SCOP1` and `SCOP2` benchmark alignments, respectively.

Following describes the nine popular amino acid substituion matrices used in this study.

PAM [10]: This is the oldest yet still widely-used Markov model which inspired many subsequent improvements. PAM was derived over a small and limited dataset, covering only 1572 substitutions (with no observed replacements for some amino acid pairs). We computed this matrix using the originally published data by Dayhoff et al. [10].

JTT [15]: This is an improved version of PAM. The main difference lies in the use of an approximate method for phylogenetic tree inference instead of the maximum parsimony method used by Dayhoff et al. [10] for obtaining substitution counts. We retrieved the JTT matrix from: `http://bioinfadmin.cs.ucl.ac.uk/downloads/Matrices`.

BLOSUM [12]: The BLOSUM series acts as a landmark, non-Markov model for substitution modelling, which is also the most used, with BLOSUM-62 being the common choice for aligning averagely distant pairs. Theoretically, any BLOSUM-n matrix can be obtained over a block of ungapped, multiple alignments. The authors originally published 16 matrices (at `ftp://ftp.ncbi.nih.gov/repository/blocks/unix/blosum` – latest version 5.0 in 1992) for $n \in \{30, 35, \ldots, 90, 95, 100\}$ and $n = 62$. They have been inferred over 2000 ungapped multiple alignment blocks from the BLOCKS database [25], across more than 500 groups of related proteins, with at least 2369 changes for each possible amino acid pair. This work took BLOSUM-62 as the representative matrix of the model.

JO [14]: This is an early, non-Markov matrix that was estimated on structural alignment data. We used the originally published substitution counts to derive the matrix (with maximum-likelihood estimates over the count data, as defined in the original paper). Their dataset covered 207,795 amino acid exchanges present in 65 homologous sets of 3D structural alignments across 235 proteins, mainly incorporating 15% to 40% sequence identity.

WAG [35]: This mainly represents substitution propensities in globular protein family sequences, inferred as a continuous-time Markov model over 3905 proteins across 182 families from an earlier existed and unpublished sequence alignment database called BRKALN. Whelan and Goldman [35] have followed an Expectation-Maximisation (EM) based maximum-likelihood (ML) method to estimate a count based rate matrix Q, by defining the likelihood of the model given the families of aligned proteins and their phylogenetic trees (with relative branch lengths), and iteratively re-inferring Q and the phylogenetic relationships until convergence. The WAG rate matrix has been published with its associated amino acid frequencies at `https://www.ebi.ac.uk/goldman-srv/WAG/wag.dat`.

VTML [24]: This stands for Variable Time Maximum Likelihood, presented as a continuous-time Markov model. Originally, Müller and Vingron [23] proposed the VT matrix, inferred via a matrix resolvent method to iteratively estimate the rate matrix over pairwise sequence alignments from the SYSTERS protein family database [18] (over 2.7 million amino acid pairs). They have used PAM as the starting point for the search. This method have also accounted for the optimal evolutionary divergence between proteins. Later, VTML was inferred using iterative ML estimation. The Perl script for generating their Markov matrix is available at: `https://owww.molgen.mpg.de/~muelle_t/vt_scores`.

LG [20]: This is an improved version of the WAG model, covering more families and accounting for rate variations across amino acid sites (through a set of gamma distributed rate categories). This incorporated 3912 seed (multiple) sequence alignments from the Pfam database [3], over around $50,000$ sequences with approximately 6.5 million amino acids. Overall, 3912 alignments with a limited number of gaps ($\sim 1\%$ of the amino acids being inserted/deleted) were involved. They also followed an EM based ML estimation similar to the procedure of WAG inference. The rate matrix and the associated amino acid frequencies are available from: `http://www.atgc-montpellier.fr/download/datasets/models/lg_LG.PAML.txt`.

MIQS [36]: This has been derived through a Principal Component Analysis (PCA) of several existing scoring matrices including BLOSUM and VTML, by sampling a new point from the PCA subspace defined by the first three principal components. The MIQS scoring matrix (published in 3 bit units) was retrieved through the DECIPHER Bioconductor package (`https://rdrr.io/bioc/DECIPHER/man/MIQS.html`).

PFASUM [16]:] This is the most recent, BLOSUM-like non-Markov family of matrices, derived over Pfam seed multiple structural alignments. Unlike BLOSUM, PFASUM has taken gapped alignments into account, while also considering special amino acids. The scoring matrix series is available at: `http://www.cbs.tu-darmstadt.de/PFASUM/`. We took PFASUM-60 as the representative matrix of the model, since the authors have recommended it to be a general choice for distant relationship detection based on their evaluations.

S4.1 Availability of Datasets and Source Code

All the above listed existing matrices abd benchmark datasets are available at our project page: `https://lcb.infotech.monash.edu/mmlsum`. We have also made our source code (C++ scripts) available at the above URL. Note: Our source code implementation utilises GNU Scientific library, openMP, Boost and other standard C++ libraries. We would also like to acknowledge the use of GNU parallel [31] when retrieving and analysing data during our experimental tasks related to this work.

References

[1] L. Allison. Vectors. In *Coding Ockham's Razor*, chapter 9, pages 89–101. Springer, 2018.

[2] A. Andreeva, E. Kulesha, J. Gough, and A. G. Murzin. The SCOP database in 2020: expanded classification of representative family and superfamily domains of known protein structures. *Nucleic Acids Research*, 48 (D1):D376–D382, 2020.

[3] A. Bateman, E. Birney, R. Durbin, S. R. Eddy, K. L. Howe, and E. L. Sonnhammer. The Pfam protein families database. *Nucleic Acids Research*, 28(1):263–266, 2000.

[4] D. M. Boulton and C. S. Wallace. The information content of a multistate distribution. *Journal of Theoretical Biology*, 23(2):269–278, 1969.

[5] N. S. Boutonnet, M. J. Rooman, M.-E. Ochagavia, J. Richelle, and S. J. Wodak. Optimal protein structure alignments by multiple linkage clustering: application to distantly related proteins. *Protein Engineering, Design and Selection*, 8(7):647–662, 1995.

[6] J. H. Collier, L. Allison, A. M. Lesk, P. J. Stuckey, M. Garcia de la Banda, and A. S. Konagurthu. Statistical inference of protein structural alignments using information and compression. *Bioinformatics*, 33(7):1005–1013, 2017. URL `https://doi.org/10.1093/bioinformatics/btw757`.

[7] U. Consortium et al. Uniprot: the universal protein knowledgebase. *Nucleic Acids Research*, 45(D1): D158–D169, 2017.

[8] J. H. Conway and N. J. Sloane. On the Voronoi regions of certain lattices. *SIAM Journal on Algebraic Discrete Methods*, 5(3):294–305, 1984.

[9] N. Daniels, A. Kumar, L. Cowen, and M. Menke. Touring protein space with Matt. *IEEE/ACM Transactions on Computational Biology and Bioinformatics*, 9(1):286–293, 2011.

[10] M. Dayhoff, R. Schwartz, and B. Orcutt. 22 a model of evolutionary change in proteins. In *Atlas of protein sequence and structure*, volume 5, pages 345–352. National Biomedical Research Foundation Silver Spring MD, 1978.

[11] A. P. Dempster, N. M. Laird, and D. B. Rubin. Maximum likelihood from incomplete data via the em algorithm. *Journal of the Royal Statistical Society: Series B (Methodological)*, 39(1):1–22, 1977.

[12] S. Henikoff and J. G. Henikoff. Amino acid substitution matrices from protein blocks. *Proceedings of the National Academy of Sciences*, 89(22):10915–10919, 1992.

[13] L. Holm and C. Sander. Protein structure comparison by alignment of distance matrices. *Journal of Molecular Biology*, 233(1):123–138, 1993.

[14] M. S. Johnson and J. P. Overington. A structural basis for sequence comparisons: an evaluation of scoring methodologies. *Journal of Molecular Biology*, 233(4):716–738, 1993.

[15] D. T. Jones, W. R. Taylor, and J. M. Thornton. The rapid generation of mutation data matrices from protein sequences. *Bioinformatics*, 8(3):275–282, 1992.

[16] F. Keul, M. Hess, M. Goesele, and K. Hamacher. Pfasum: a substitution matrix from PFAM structural alignments. *BMC Bioinformatics*, 18(1):293, 2017.

[17] S. Kirkpatrick, C. D. Gelatt, and M. P. Vecchi. Optimization by simulated annealing. *Science*, 220(4598): 671–680, 1983.

[18] A. Krause, J. Stoye, and M. Vingron. The SYSTERS protein sequence cluster set. *Nucleic Acids Research*, 28(1):270–272, 2000.

[19] S. Kullback and R. A. Leibler. On information and sufficiency. *The Annals of Mathematical Statistics*, 22 (1):79–86, 1951.

[20] S. Q. Le and O. Gascuel. An improved general amino acid replacement matrix. *Molecular Biology and Evolution*, 25(7):1307–1320, 2008.

[21] M. Menke, B. Berger, and L. Cowen. Matt: local flexibility aids protein multiple structure alignment. *PLoS Computational Biology*, 4(1):e10, 2008.

[22] K. Mizuguchi, C. Deane, T. Blundell, and J. Overington. HOMSTRAD: a database of protein structure alignments for homologous families. *Protein Science*, 7(11):2469–2471, 1998.

[23] T. Müller and M. Vingron. Modeling amino acid replacement. *Journal of Computational Biology*, 7(6): 761–776, 2000.

[24] T. Müller, R. Spang, and M. Vingron. Estimating amino acid substitution models: a comparison of Dayhoff's estimator, the resolvent approach and a maximum likelihood method. *Molecular Biology and Evolution*, 19(1):8–13, 2002.

[25] S. Pietrokovski, J. G. Henikoff, and S. Henikoff. The blocks database—a system for protein classification. *Nucleic Acids Research*, 24(1):197–200, 1996.

[26] R. B. Russell and G. J. Barton. Multiple protein sequence alignment from tertiary structure comparison: assignment of global and residue confidence levels. *Proteins: Structure, Function, and Bioinformatics*, 14 (2):309–323, 1992.

[27] A. Sali and T. Blundell. The definition of topological equivalence in homologous and analogous structures: A procedure involving comparison of local properties and structural relationships through dynamic programming and simulated annealing. *Journal of Molecular Biology*, 212:403–428, 1990.

[28] I. N. Shindyalov and P. E. Bourne. Protein structure alignment by incremental combinatorial extension (CE) of the optimal path. *Protein Engineering*, 11(9):739–747, 1998.

[29] D. Sumanaweera, L. Allison, and A. S. Konagurthu. Statistical compression of protein sequences and inference of marginal probability landscapes over competing alignments using finite state models and Dirichlet priors. *Bioinformatics*, 35(14):i360–i369, 2019.

[30] M. J. Sutcliffe, I. Haneef, D. Carney, and T. Blundell. Knowledge based modelling of homologous proteins, Part I: Three-dimensional frameworks derived from the simultaneous superposition of multiple structures. *Protein Engineering, Design and Selection*, 1(5):377–384, 1987.

[31] O. Tange et al. Gnu parallel-the command-line power tool. *The USENIX Magazine*, 36(1):42–47, 2011.

[32] I. Van Walle, I. Lasters, and L. Wyns. SABmark—a benchmark for sequence alignment that covers the entire known fold space. *Bioinformatics*, 21(7):1267–1268, 2005.

[33] C. S. Wallace. *Statistical and inductive inference by minimum message length.* Science & Business Media. Springer, 2005.

[34] C. S. Wallace and P. R. Freeman. Estimation and inference by compact coding. *Journal of the Royal Statistical Society. Series B (Methodological)*, pages 240–265, 1987.

[35] S. Whelan and N. Goldman. A general empirical model of protein evolution derived from multiple protein families using a maximum-likelihood approach. *Molecular Biology and Evolution*, 18(5):691–699, 2001.

[36] K. Yamada and K. Tomii. Revisiting amino acid substitution matrices for identifying distantly related proteins. *Bioinformatics*, 30(3):317–325, 2013.
